# Genomic characterization of *Enterobacter* isolates highlights widespread ST78 high-risk clone and plasmid-mediated dissemination of *bla*_NDM-1_

**DOI:** 10.1128/spectrum.04019-25

**Published:** 2026-06-15

**Authors:** Luis Ángel Núñez-García, Elvira Garza-González, Claudia Adriana Colín-Castro, María Guadalupe Martinez-Zavaleta, Humberto Torres-Rodríguez, Christian Daniel Mireles-Davalos, Rosario Vázquez-Larios, Sandra Quintana-Ponce, Victor Antonio Monroy-Colin, Cristoforo Alejandro Gómez-Quiroz, Juan de Dios Castañeda-Duarte, Enrique Bolado-Martínez, María del Rocío Vazquez-Olivares, Maria del Consuelo Velazquez-Acosta, Juan Manuel Barajas-Magallón, Hiram Villanueva-Lozano, Talia Pérez-Vicelis, Eloisa Ramírez-Alanis, Dora Elia Rodriguez-Balderas, Eduardo López-Gutiérrez, Maribel López-Garcia, Cecilia Padilla-Ibarra, Mariana Gil-Veloz, Carlos Antonio Couoh-May, Alondra Aguilar-Sandoval, Juan Pablo Mena-Ramírez, Javier Paul Mora-Domínguez, Elena Victoria Choy-Chang, Daniel Romero-Romero, Ulises Garza-Ramos, Manuel Poblano-Morales, Luis Esaú López-Jácome

**Affiliations:** 1 Departamento de Bioquímica y Medicina Molecular, Universidad Autónoma de Nuevo León 27771, Monterrey, Nuevo León, Mexico; 2 Instituto Nacional de Rehabilitación Luis Guillermo Ibarra Ibarra 61663 , Mexico City, Mexico; 3 Instituto Nacional de Enfermedades Respiratorias 42635 , Mexico City, Mexico; 4 Instituto Nacional de Cardiología Ignacio Chávez 61588 , Mexico City, Mexico; 5 Universidad Autónoma de Guerrero 27768, Guerrero, Mexico; 6 Centenario Hospital Miguel Hidalgo, Aguascalientes, Mexico; 7 Hospital Civil de Guadalajara Fray Antonio Alcalde 103531 , Jalisco, Mexico; 8 Centro Médico Doctor Ignacio Chávez ISSSTESON, Sonora, Mexico; 9 Universidad de Sonora 27813, Sonora, Mexico; 10 Centro Nacional de Referencia de Inocuidad y Bioseguridad Agroalimentaria, Mexico City, Mexico; 11 Instituto Nacional de Cancerología 42597 , Mexico City, Mexico; 12 Laboratorio DIPROMI, Morelia, Mexico; 13 Hospital Regional "Alta Especialidad" ISSSTE Monterrey, Nuevo León, Mexico; 14 Hospital Regional de Alta Especialidad Bicentenario de la Independencia ISSSTE Tultitlán, Mexico City, Mexico; 15 Hospital la Luz Morelia, Morelia, Mexico; 16 Hospital de Niño Dr. Federico Gómez Santos, Coahuila de Zaragoza, Mexico; 17 Hospital Regional de Alta Especialidad de Oaxaca 126672 , Oaxaca, Mexico; 18 Hospital de la Madre y el Niño Guerrerense, Guerrero, Mexico; 19 Hospital General del Estado de Sonora 385683 , Sonora, Mexico; 20 Hospital Regional de Alta Especialidad del Bajío 126671, Guanajuato, Mexico; 21 Hospital General Dr. Agustin O'Horan 162251 , Yucatan, Mexico; 22 Hospital General de León, Guanajuato, Mexico; 23 Hospital General de Zona 21 Tepatitlán de Morelos. Centro Universitario de los Altos (CUALTOS), Universidad de Guadalajara 56076 , Tepatitlán de Morelos, Mexico; 24 Hospital de Alta Especialidad de Veracruz 665579 , Veracruz, Mexico; 25 Hospital General de Zona No. 1 42564, Tapachula, Chiapas, Mexico; 26 Diagnóstico Médico Integral Pasteur, Mexico City, Mexico; 27 Instituto Nacional de Salud Publica 37764, Cuernavaca, Morelos, Mexico; 28 Hospital HMAS Queretaro by Hospitales MAC, Queretaro, Mexico; 29 Instituto Nacional de Rehabilitacion Luis Guillermo Ibarra Ibarra 61663 , Mexico City, Mexico; University of Guelph College of Biological Science, Guelph, Ontario, USA

**Keywords:** *Enterobacter*, genomic epidemiology, carbapenemases, plasmid-mediated resistance, antibiotic resistance

## Abstract

Species of the genus *Enterobacter* are considered priority pathogens due to their extensive antimicrobial resistance, primarily driven by transmissible β-lactamases. We conducted whole-genome sequencing of clinical isolates of *Enterobacter* spp. collected between 2020 and 2024 across 14 federal entities in Mexico. Subspecies-level identification revealed the predominance of *Enterobacter hormaechei* subsp. *steigerwaltii* and *E. hormaechei* subsp. *xiangfangensis*, the latter showing the highest resistance rates to tested antimicrobial agents. Resistome profiling identified *bla*_NDM-1_ as the most prevalent carbapenemase among carbapenem-resistant isolates. Mobilome analysis showed that *bla*_NDM-1_ was predominantly carried by plasmids of the IncFIB/IncFII/rep_cluster_2272 type. Phylogenomic comparisons with public genomes, combined with the dominance of *bla*_NDM-1_, suggest transcontinental dissemination. Notably, international clones of *E. hormaechei* subspecies, such as ST182 and ST78, were identified, alongside regionally relevant clones such as ST92, ST45, ST116, and ST93. Finally, genomic evidence supports the existence of a putative novel *Enterobacter* species related to *Enterobacter bugandensis*, *Enterobacter nematophilus*, and *Enterobacter chuandaensis*.

**IMPORTANCE:**

*Enterobacter* species pose a global threat due to their capacity to acquire and disseminate antimicrobial resistance. Currently, there is no genomic surveillance data from Mexico, limiting the understanding of the national molecular epidemiology and its connection with global trends. This study provides the first nationwide genomic overview of *Enterobacter* in Mexico. Our findings highlight the detection of both localized and widespread clones, including a recognized high-risk lineage, and dissemination of *bla*_NDM_. Beyond public health implications, we describe a putative novel species. By making this genomic data publicly available, this work contributes to the generation of data needed for surveillance, outbreak detection, and strategies to control antimicrobial resistance in *Enterobacter* species.

## INTRODUCTION

Species of the genus *Enterobacter* are listed in the critical group of the World Health Organization bacterial priority pathogens list (1). Of the 22 recognized *Enterobacter* species, members of the *Enterobacter cloacae* complex (ECC) are the most frequently isolated in clinical settings (2). Infectious diseases caused by *Enterobacter* spp. resistant to third-generation cephalosporins and carbapenems are associated with mortality rates exceeding 30% (1). All these species have distinct antimicrobial resistance (AMR) profiles and virulence; therefore, accurate taxonomic identification is needed (3, 4).

Antibiotic-degrading enzymes are the main AMR mechanism in the ECC (2), particularly the production of extended-spectrum β-lactamases (ESBLs) and carbapenemases. ESBLs and carbapenemase-encoding genes may be located on the chromosome or plasmids. The most common ESBL alleles belong to the *bla*_TEM_, *bla*_SHV_, and *bla*_CTX-M_ families (2, 5–7). Carbapenemase genes mainly include the *bla*_NDM_, *bla*_VIM_, *bla*_KPC_, and *bla*_OXA_ families (8–10) and show a geographically stratified family distribution: *bla*_NDM_ in Asia, *bla*_VIM_ in Europe, and *bla*_KPC_ in America (11). The dissemination of carbapenemase genes mainly relies on plasmids, and numerous replicon types have been reported as carriers of these genes (2, 5, 6).

The ECC encodes numerous virulence factors (VFs), most of which have been characterized in *E. cloacae sensu stricto*. Among these VFs, the Type VI secretion system (T6SS) is involved in colonization and biofilm formation (12–14). Furthermore, genes associated with iron acquisition, adhesion, and toxin production are also present, albeit at lower frequencies (13).

Despite the relevance of the *Enterobacter* genus, genomic data from America, and particularly from Mexico, remain scarce, limiting the understanding of the regional molecular epidemiology, resistance mechanisms, virulence properties, and clonal dynamics of these pathogens. In this study, we conducted a nationwide, multicenter genomic surveillance study of *Enterobacter* isolates collected from healthcare centers between 2020 and 2024.

## MATERIALS AND METHODS

### Clinical isolates, identification, susceptibility testing, and carbapenemase production

Clinical isolates belonging to the *Enterobacter* genus and initially identified as multi-drug resistant (MDR) (15) collected between 2020 and 2024 from 24 healthcare centers that are members of the INVIFAR network (Red Temática de Investigación y Vigilancia de la Farmacorresistencia, https://invifar.ucol.mx/) across 14 Federal Entities were included (Table S1). All clinical isolates were recovered as potentially causative agents of infection and sent to the coordinating laboratory.

Species identification and antibiotic susceptibility were determined using the VITEK 2 system (bioMérieux, Marcy-l'Étoile, France) and confirmed by matrix-assisted laser desorption/ionization time-of-flight mass spectrometry (VITEK MS, bioMérieux). Results were interpreted using CLSI M100, 35th edition. Carbapenemase and metallo-β-lactamase production were tested using the modified carbapenem inactivation method (mCIM) and mCIM in the presence of EDTA (eCIM) according to CLSI guidelines (16). Isolates were cryopreserved at −80°C in trypticase soy broth with 15% glycerol until used.

### DNA isolation and whole-genome sequencing

Frozen isolates were thawed and cultured overnight on MacConkey agar at 37°C. Genomic DNA was extracted using the phenol-chloroform method with previous enzymatic digestion with lysozyme (750 μg/mL; 37°C, 2 h) and proteinase K (150 μg/mL; 55°C, 2 h); the purified material was resuspended in TE buffer. DNA concentration and quality were determined using a Qubit 4.0 fluorometer (Invitrogen, USA). Library preparation was performed using the Illumina DNA Prep Kit (Illumina, USA). Whole-genome sequencing was performed using the Illumina NextSeq 550 platform under a paired-end configuration.

### Genome assembly and annotation

Sequencing reads underwent quality and adapter filtering using Fastp (17), and *de novo* genome assembly was carried out with Unicycler (18). Assembly quality was assessed with QUAST (19) and CheckM (20); annotation was performed using Prokka (21).

### Species assignment and multi-locus sequence typing

Four approaches were used to perform species-level identification. First, reference genomes from the *Enterobacter* genus were retrieved from NCBI, and average nucleotide identity (ANI) was calculated using ANIclustermap (22); ANI values of 96% were initially considered for defining species boundaries (23). Second, core genome alignments were generated with Panaroo (24) and used to construct a maximum-likelihood (ML) phylogenetic tree using IQ-TREE (25) with the General Time Reversible substitution model, and branch support was assessed by 1,000 ultrafast bootstrap replicates. For the third approach, ribosomal multi-locus sequence typing (rMLST) was performed using the PubMLST RESTful API (26). Finally, isolates with inconclusive taxonomy were submitted to the Type Strain Genome Server (27) for additional resolution. Sequence types (STs) were determined using the MLST command-line tool (28), and allele profiles were compared by performing goeBURST analysis in the PhyloViZ web server (https://online.phyloviz.net).

### Resistome, mobilome, and virulome

ABRicate (29) was used to identify AMR and VF genes. The default database from the CARD (30) was used for AMR genes and a custom database using the VFDB data set (31) for VF genes. To analyze genes associated with colistin resistance, sequences of *mgrB, PhoPQ*, and *arnBCADTEF* were extracted from the annotated proteomes from NCBI and aligned using MAFFT (32). Mobile genetic elements were screened using Mobile Element Finder (33). Plasmid sequences were identified by aligning raw reads to reference genomes using Snippy (34), performing assembly of unmapped reads with Unicycler (18), and reconstructing sequences using MOB-suite (35) (coverage >80%). Selected plasmids were annotated and visualized using Proksee (36) and Clinker (37). The plasmid sequences were individually screened for AMR and VF genes using the previously described approach. Genes were considered to be of plasmid origin if they were identified both in the draft genome and in the reconstructed plasmids. Data were corroborated using the fully integrated web-based software application EPISEQ CS v2.4.10 (bioMérieux).

### Phylogenetic analysis and comparison with public records

Assemblies were compared to public *Enterobacter* genomes retrieved from the NCBI RefSeq database (*n* = 7,906) using Sourmash (38); the five most similar genomes for each isolate were selected for further analysis, and duplicate RefSeq genomes were filtered out. The pangenome of the full data set was constructed using Panaroo to generate core genome alignment and analyzed by maximum-likelihood phylogeny using IQ-TREE. The best-fit substitution for each gene within the alignment was determined using the Model Finder module, and branch support was assessed via 1,000 ultrafast bootstrap replicates.

### Statistical analysis

Comparison of the average number of AMR and VF genes across *Enterobacter* species was performed using the Kruskal-Wallis test. Differences in the overall composition of AMR and VF genes were evaluated using permutational multivariate analysis of variance. For comparisons between invasive vs non-invasive isolates, the Student’s *t*-test was applied when data followed a normal distribution previously determined by the Shapiro-Wilk test. A *P*-value < 0.05 was considered statistically significant in all tests. All statistical analyses were conducted in R (https://www.r-project.org/).

## RESULTS

### Clinical isolates and sample origin

A total of 122 isolates from diverse clinical sources classified as invasive and non-invasive were analyzed (Table S2). Invasive specimens included blood (*n* = 26), biopsies (*n* = 11), drainage fluids (*n* = 3), cerebrospinal fluid (*n* = 1), abscesses (*n* = 1), and pleural fluid (*n* = 1). Non-invasive specimens comprised urine (*n* = 49), lower respiratory samples (*n* = 10), wounds (*n* = 16), and others (*n* = 4).

### Genome assembly

Quality statistics are shown in Table S3. Assemblies had an average completeness of 99.65%, with a minimum value of 97.39%; the average contamination was 0.64%, with a maximum value of 1.87%.

### Species assignment and molecular epidemiology

**Fig 1:**
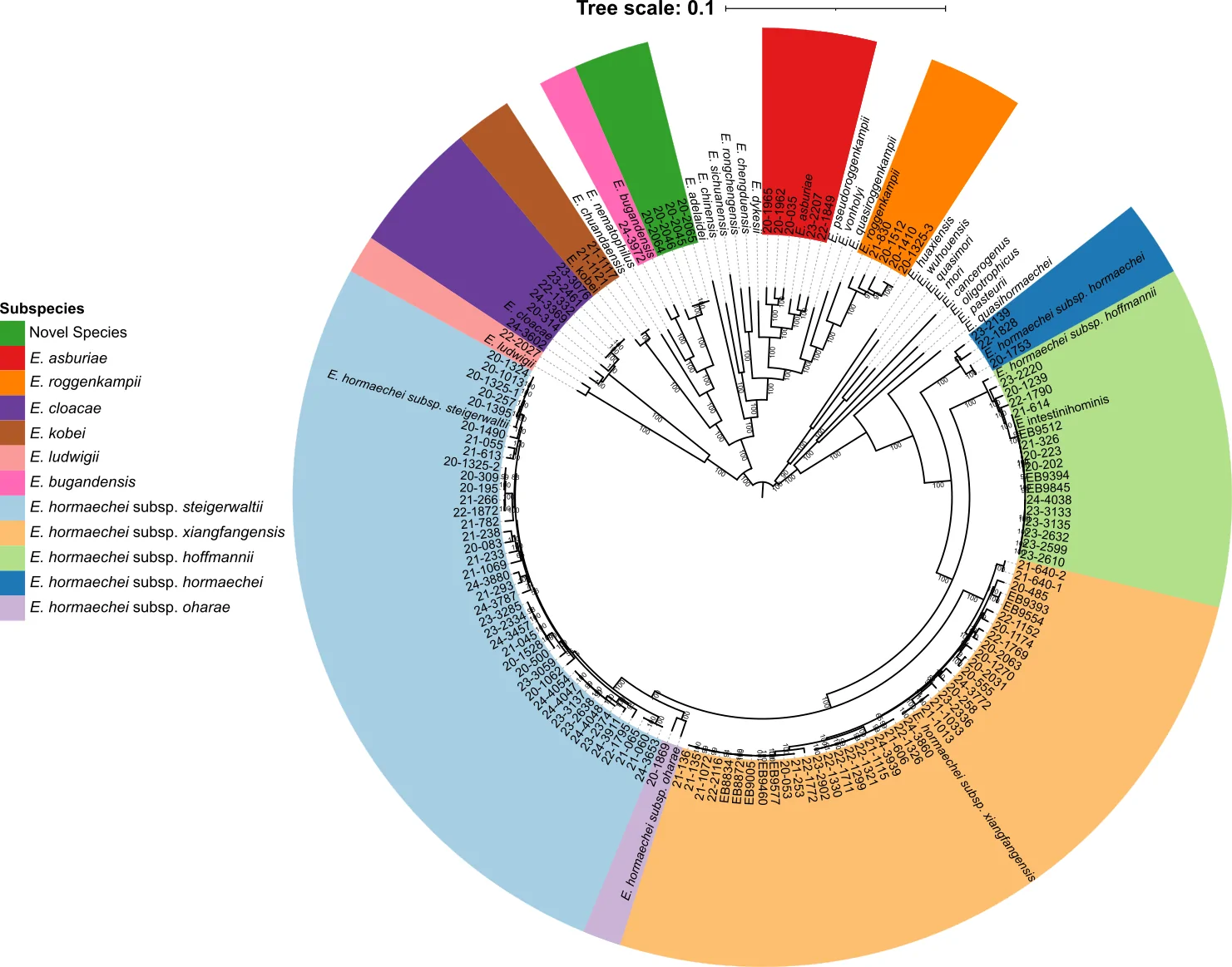
Midpoint-rooted core genome maximum-likelihood phylogenetic tree. Core genome alignments for assemblies of this study and representative genomes from the *Enterobacter* genus were used to generate a maximum-likelihood phylogenetic tree. Colors depict the species assignment chosen for this study.

Four isolates (20-2045, 20-2046, 20-2065, and 20-2064) showed discrepancies among the species identification approaches used. ANI values (~95%) indicated close relatedness to *Enterobacter nematophilus* (Fig. S1), whereas rMLST assigned them as *Enterobacter kobei* with a low confidence value (~16%) (Table S2). In contrast, core-genome phylogeny and results from the TYGS grouped these isolates into a distinct clade, suggesting that they represent a novel species within the *Enterobacter* genus (Fig. 1; Fig. S2). Furthermore, isolates identified as *Enterobacter hormaechei* subsp. *hoffmannii* (*n* = 16), along with the corresponding reference genome, clustered with *Enterobacter intestinihominis* across all species assignment strategies. Given the recent description of *E. intestinihominis* as a novel species (39) and the results obtained in this study, these isolates are referred to as *E. hormaechei* subsp. *hoffmannii* (Fig. 1; Fig. S1 and S3).

*Enterobacter hormaechei* was the most frequent species (*n* = 99), distributed among the following subspecies: *E. hormaechei* subsp. *steigerwaltii* (*n* = 40), *E. hormaechei* subsp. *xiangfangensis* (*n* = 39), *E. hormaechei* subsp. *hoffmannii* (*n* = 16), *E. hormaechei* subsp. *hormaechei* (*n* = 3), and *E. hormaechei* subsp. *oharae* (*n* = 1). Other species included *E. cloacae* (*n* = 6), *Enterobacter asburiae* (*n* = 5), *Enterobacter roggenkampii* (*n* = 4), the putative novel species (*n* = 4), *E. kobei* (*n* = 2), *Enterobacter bugandensis* (*n* = 1), and *Enterobacter ludwigii* (*n* = 1) (Fig. 1).

**Fig 2:**
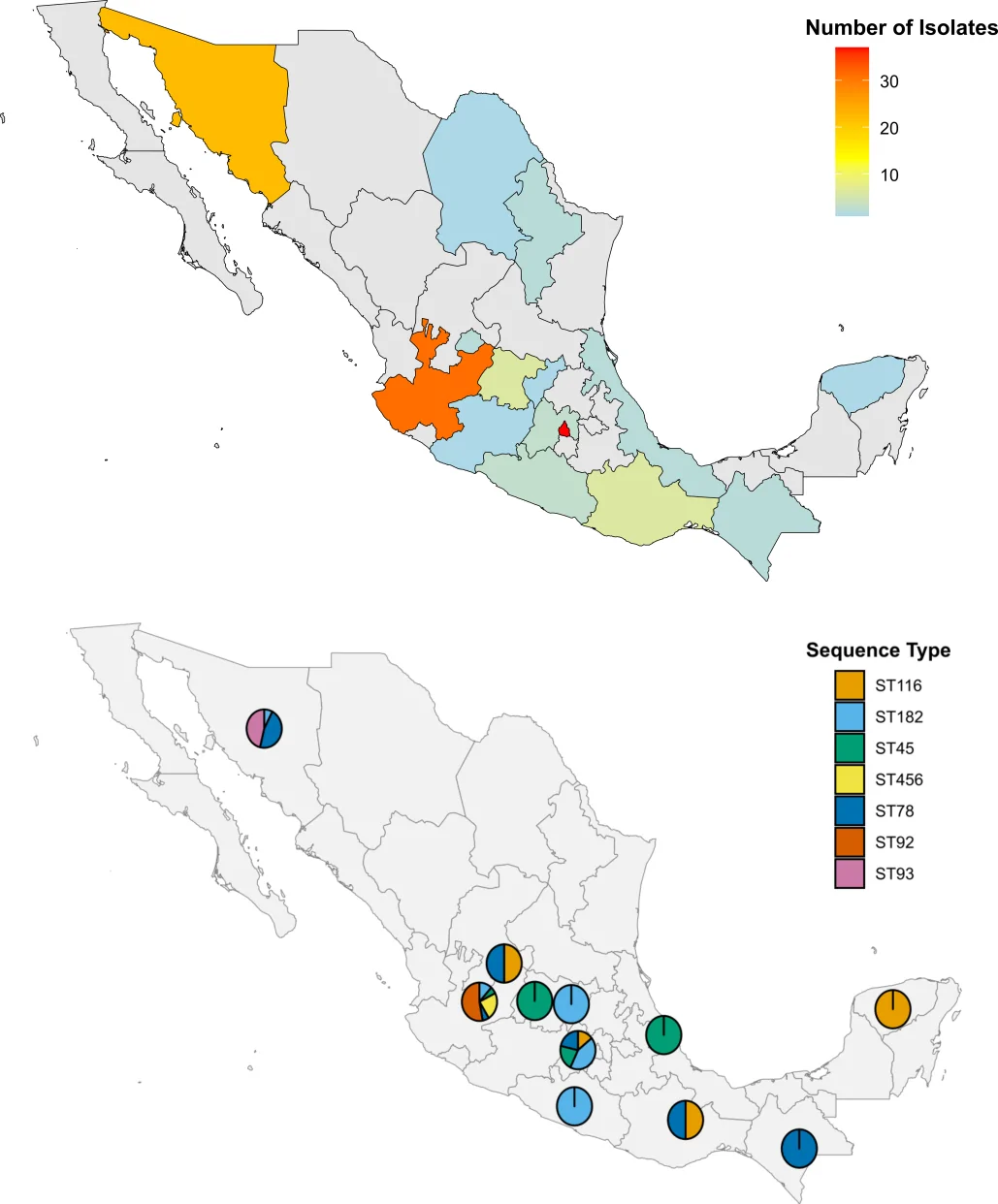
Isolates by federal entity (top) and distribution of the most frequent sequence types (bottom). The maps were generated using the public domain R package “rnaturalearth” (v1.2.0) (https://cran.r-project.org/web/packages/rnaturalearth/vignettes/rnaturalearth.html).

MLST distribution across species and federal entities is shown in Table 1 and Fig. 2. *E. hormaechei* subsp. *steigerwaltii* (*n* = 40) comprised 21 STs, with ST45 (*n* = 6, five federal entities), ST93 (*n* = 6, one federal entity), and ST116 (*n* = 5, four federal entities) being the most frequent. Fifteen STs (37.5%) were detected once. *E. hormaechei* subsp. *xiangfangensis* isolates (*n* = 39) were represented by 16 STs, of which ST182 (*n* = 11, five federal entities) and ST92 (*n* = 9, one federal entity) were the most frequent, and 10 STs (25.64%) occurred once. *E. hormaechei* subsp. *hoffmannii* (*n* = 16) primarily belonged to ST78 (*n* = 13, six federal entities); three STs were identified once. The remaining species consisted of one representative ST and STs of unique occurrence. Fifteen isolates had novel MLST profiles; upon curation, 11 new profiles were assigned (Table 1). Five single-locus variant pairs were identified by goeBURST analysis: ST98-ST182, ST1772-ST2734, ST114-ST418, ST45-ST972, and ST488-ST3390. The remaining STs were classified as singletons, since they differ in two or more MLST alleles from all STs in this data set (Fig. S4).

**TABLE 1:** Distribution of STs across species of the included *Enterobacter* spp. isolates^*a*^

Species	MLST	Count (%)	STs of unique occurrence
*E. hormaechei* subsp. *steigerwaltii* (*n* = 40)	ST45	6 (15.00)	ST50, ST89, ST133, ST146, ST184, ST346, ST488, ST536, ST604, ST972, ST981, **ST3390, ST3391, ST3392, ST3393**
	ST93	6 (15.00)	
	ST116	5 (12.50)	
	ST91	3 (7.50)	
	**ST3396**	3 (7.50)	
	ST426	2 (5.00)	
	Unique	15 (37.50)	
*E. hormaechei* subsp. x*iangfangensis* (*n* = 39)	ST182	11 (28.21)	ST63, ST98, ST114, ST136, ST171, ST418, ST459, ST1772, ST2734, **ST3394**
	ST92	9 (23.08)	
	ST544	3 (7.69)	
	ST109	2 (5.13)	
	ST264	2 (5.13)	
	ST270	2 (5.13)	
	Unique	10 (25.64)	
*E. hormaechei* subsp. *hoffmannii* (*n* = 16)	ST78	13 (81.25)	ST145, ST173, ST233
	Unique	3 (18.75)	
*E. hormaechei* subsp. *hormaechei* (*n* = 3)	Unique	3 (100.00)	ST528, ST1749, ST1848
*E. hormaechei* subsp. *oharae* (*n* = 1)	Unique	1 (100.00)	ST68
*E. cloacae* (*n* = 6)	ST456	4 (66.67)	**ST3389, ST3399**
	Unique	2 (33.33)	
*E. asburiae* (*n* = 5)	Unique	5 (100.00)	ST24, ST25, ST162, ST733, **ST3398**
Novel Species (*n* = 4)	**ST3388**	3 (75.00)	**ST3395**
	Unique	1 (25.00)	
*E. roggenkampii* (*n* = 4)	ST2271	2 (50.00)	ST272, ST826
	Unique	2 (50.00)	
*E. kobei* (*n* = 2)	ST54	2 (100.00)	N/A
*E. ludwigii* (*n* = 1)	Unique	1 (100.00)	ST3041
*E. bugandensis* (*n* = 1)	Unique	1 (100.00)	ST2268

^*a*^ MLST, multi-locus sequence typing; ST, sequence type; and N/A, non-applicable. Bold values indicate novel sequence types.

### Antibiotic susceptibility testing

Overall resistance rates are shown in Table S4; the highest resistance rate was observed for ceftazidime (73.3%), followed by ertapenem (58.3%). Within species, resistance rates of *E. hormaechei* subsp. *xiangfangensis* isolates were 74.3% (representing 23.7% of all isolates) for ciprofloxacin and 61.5% (19.6% of the total) for ceftazidime/avibactam and imipenem, surpassing those observed in the other subspecies of *E. hormaechei*. For *E. cloacae* (*n* = 6)*,* resistance rates were >60% (3.2% of the total) to the majority of the tested antimicrobial agents. Resistance to colistin was detected in *E. hormaechei* subsp. *steigerwaltii* (1/40, 2.5%; 0.8% of the total), *E. cloacae* (4/6, 66.6%; 3.2% of the total), *E. asburiae* (2/5, 40.0%; 1.6% of the total), in the putative novel species (4/4, 100%, 3.2% of the total), *E. roggenkampii* (1/4, 25%; 0.8% of the total), *E. kobei* (1/2, 50%; 0.8% of the total), and *E. bugandensis* (1/1, 100%; 0.8% of the total) (Table 2).

**TABLE 2:** Antibiotic resistance rates among the tested *Enterobacter* spp. isolates^*a*^

Species	TZP	CAZ	CZA	CTX	FEP	ATM	ETP	IPM	MEM	AMK	CIP^*b*^	TGC^*b*^	LVX^*b*^	GEN^*b*^	TOB^*b*^	SXT^*b*^	CST^*b*^
*E. hormaechei* subsp. *steigerwaltii* (*n* = 40)	31 (77.5)	27 (67.5)	13 (32.5)	27 (67.5)	18 (45)	25 (62.5)	24 (60)	12 (30)	15 (37.5)	19 (47.5)	20 (50)	18 (45)	17 (42.5)	17 (42.5)	14 (35)	16 (40)	1 (2.5)
*E. hormaechei* subsp. *xiangfangensi* (*n* = 39)	35 (89.7)	34 (87.1)	24 (61.5)	31 (79.4)	28 (71.7)	33 (84.6)	27 (69.2)	24 (61.5)	25 (64.1)	24 (61.5)	29 (74.3)	19 (48.7)	17 (43.5)	26 (66.6)	30 (76.9)	29 (74.3)	0
*E. hormaechei* subsp. *hoffmannii* (*n* = 16)	15 (93.7)	14 (87.5)	6 (37.5)	14 (87.5)	13 (81.2)	13 (81.2)	10 (62.5)	7 (43.7)	6 (37.5)	12 (75)	14 (87.5)	7 (43.7)	9 (56.2)	12 (75)	12 (75)	8 (50)	0
*E. hormaechei* subsp. *hormaechei* (*n* = 3)	3 (100)	3 (100)	1 (33.3)	1 (33.3)	1 (33.3)	3 (100)	1 (33.3)	1 (33.3)	1 (33.3)	0	0	0	0	0	0	3 (100)	0
*E. hormaechei* subsp. *oharae* (*n* = 1)	0	0	0	0	0	0	0	0	0	0	0	0	0	0	0	0	0
*E. cloacae* (*n* = 6)	6 (100)	6 (100)	4 (66.6)	6 (100)	4 (66.6)	6 (100)	5 (83.3)	4 (66.6)	4 (66.6)	4 (66.6)	4 (66.6)	4 (66.6)	3 (50)	4 (66.6)	5 (83.3)	4 (66.6)	4 (66.6)
*E. asburiae* (*n* = 5)	2 (40)	2 (40)	1 (20)	2 (40)	1 (20)	0	1 (20)	2 (40)	2 (40)	1 (20)	1 (20)	0	0	0	2 (40)	1 (20)	2 (40)
Novel Species (*n* = 4)	0	0	0	0	0	0	0	0	0	0	0	0	0	0	0	0	4 (100)
*E. roggenkampii* (*n* = 4)	1 (25)	1 (25)	0	1 (25)	0	1 (25)	0	0	0	0	2 (50)	3 (75)	2 (50)	0	0	3 (75)	1 (25)
*E. kobei* (*n* = 2)	2 (100)	2 (100)	2 (100)	2 (100)	1 (50)	0	1 (50)	2 (100)	2 (100)	2 (100)	0	1 (50)	0	0	0	2 (100)	1 (50)
*E. ludwigii* (*n* = 1)	1 (100)	0	0	0	0	0	1 (100)	0	0	0	1 (100)	0	0	0	0	0	0
*E. bugandensis* (*n* = 1)	0	0	0	0	0	0	0	0	0	0	0	0	0	0	1 (20)	0	0

^*a*^ TZP, piperacillin-tazobactam; CAZ, ceftazidime; CZA, ceftazidime-avibactam; CTX, cefotaxime; FEP, cefepime; ATM, aztreonam; ETP, ertapenem; IPM, imipenem; MEM, meropenem; AMK, amikacin; CIP, ciprofloxacin; TGC, tigecycline; LVX, levofloxacin; GEN, gentamicin; TOB, tobramycin; SXT, trimethoprim-sulfamethoxazole; and CST, colistin. Percentages are indicated in parentheses. Tigecycline susceptibility was interpreted according to EUCAST version 15.

^*b*^ Tested using the Kirby-Bauer disk diffusion method.

### Resistome

**Fig 3:**
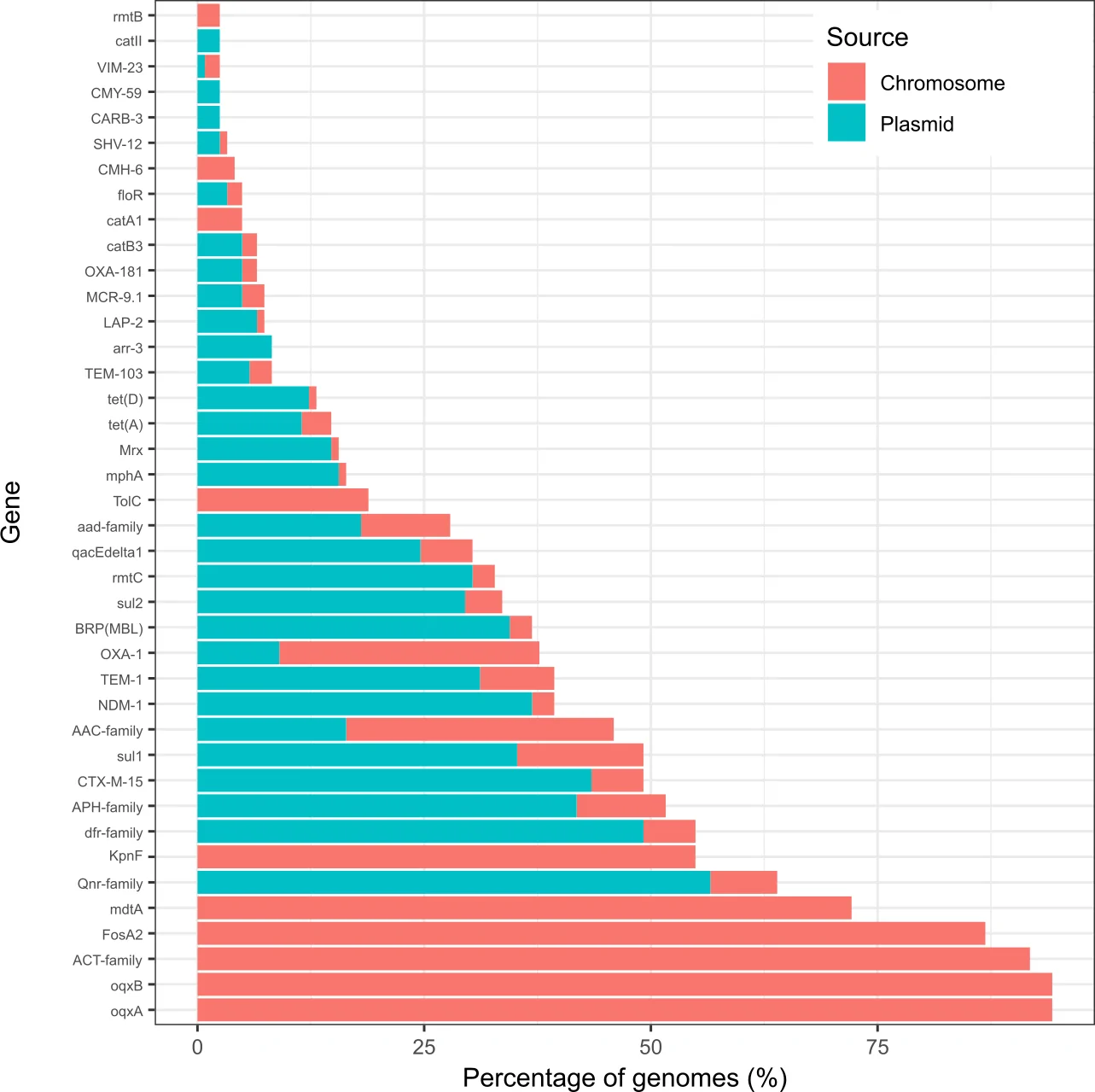
Frequency of antimicrobial resistance genes according to their source.

Isolates harbored an average of 12 AMR genes. The number of AMR genes did not vary significantly between species (*P* > 0.05). Aside from core AMR determinants, genes from the *oqxA*, *oqxB*, *bla*_ACT_, and *fosA2* families were detected in ~90% of isolates and were exclusively of chromosome origin (Fig. 3). Genes encoding quinolone resistance proteins (*Qnr*; 63.9%), trimethoprim resistance enzymes (*dfr*; 54.9%), aminoglycoside phosphotransferases (*APH*; 51.6%), sulfonamide thiol transferases (*sul1*; 49.1%), and *bla*_CTX-M-15_ (49.1%) were detected in ~50% of isolates and were mostly plasmid-borne.

Resistome profiling using ABRicate identified 59 (48.3%) isolates with at least one carbapenemase, of which 55 (45.0%) were resistant to at least one carbapenem. The most common carbapenemase was *bla*_NDM-1_, detected in 48 (39.3%) isolates, of which 47 (38.5%) were resistant to at least two carbapenems and positive for mCIM and eCIM. Isolates identified as *E. hormaechei* subsp. *xiangfangensis* and *E. hormaechei* subsp. *steigerwaltii* accounted for most of the *bla*_NDM-1_-positive isolates (*n* = 23 and *n* = 11, respectively) (Table S5). Eight isolates harbored *bla*_OXA-181_, six of them were resistant to at least one carbapenem; five of these were identified as *E. hormaechei* subsp. *steigerwaltii*. Three isolates were positive for *bla*_VIM-23_, two of which were identified as *E. hormaechei* subsp. *xiangfangensis* and one as *E. asburiae*; these strains were carbapenem-resistant and positive for mCIM/eCIM. The *mcr-9.1* gene was detected in nine clinical isolates, and one of them was colistin-resistant (Table S5); three of these isolates co-harbored *bla*_NDM_.

**Fig 4:**
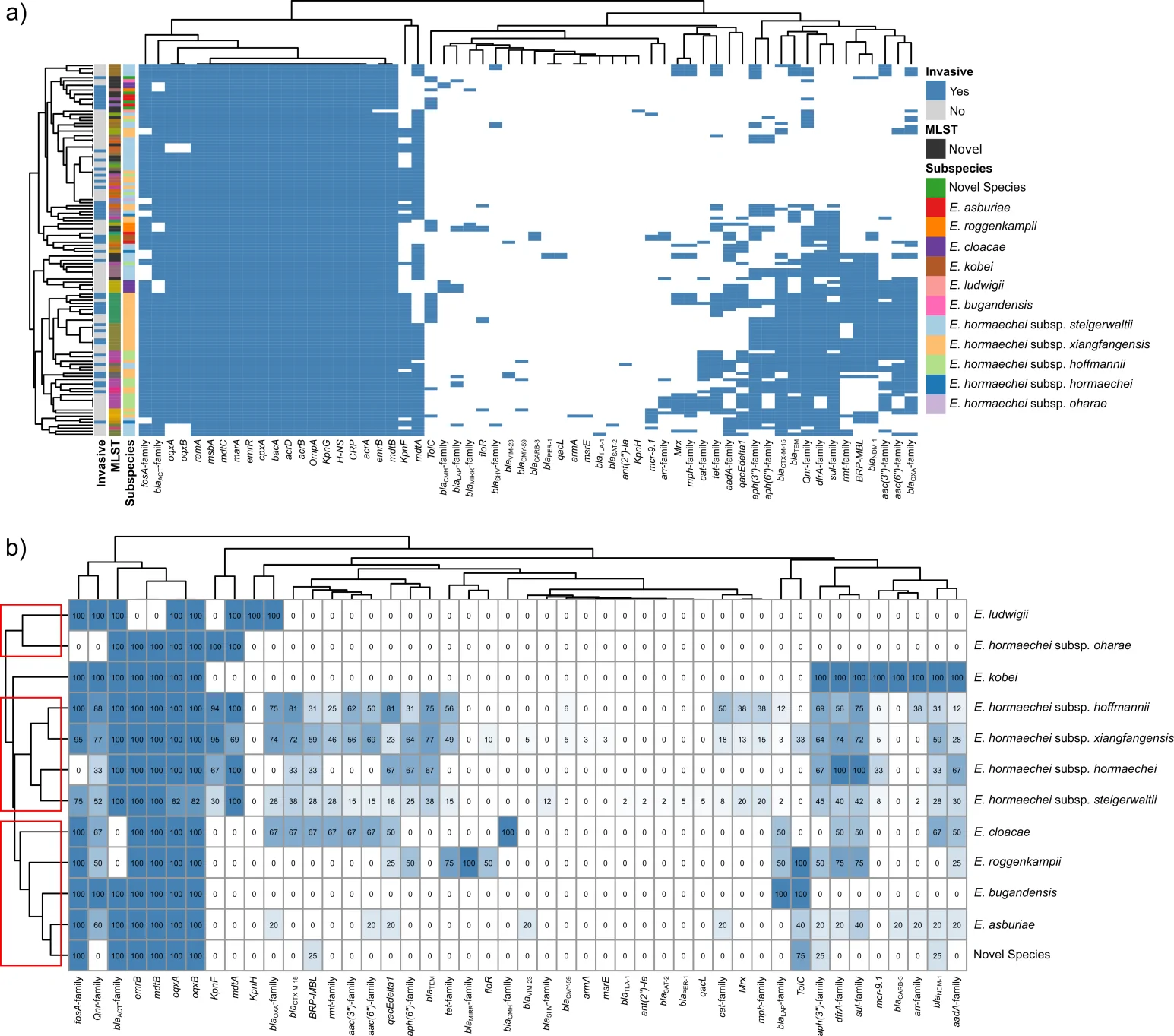
Resistome profiles of the sequenced isolates. (**a**) Distribution of resistance genes across all the included isolates. Only novel sequence types are included in the legend for visual purposes. (**b**) Resistance gene frequency distribution by species and subspecies; numbers inside the boxes indicate the percentage of isolates of the corresponding species (rows) with the gene (columns). Genes with a frequency >98% (core genes) are not shown for simplicity.

*bla*_CMY_, *bla*_PER_, *bla*_SHV_, *bla*_TEM_, *bla*_TLA_, macrolide phosphotransferases (*mph*), small multidrug efflux pumps (*qacG2*/*qacL*), and tetracycline efflux pumps (*tetABCD*) (30) were only detected among subspecies of *E. hormaechei. bla*_MIRR_ was only detected in isolates of *E. roggenkampii*, and *bla*_CMH_ was unique to *E. cloacae* isolates (Fig. 4).

ADP-ribosyltransferases (*arr* family) (*P* = 0.013) and *kpnF* (*P* = 0.016) were more frequent in *E. hormaechei* subsp. *hoffmannii* and *E. hormaechei* subsp. *xiangfangensis*. The outer membrane protein gene *tolC* was significantly enriched in both *E. roggenkampii* and the putative novel species (*P* = 0.014) (Fig. 4b). Four main clusters were observed in the AMR gene matrix (Fig. 4b), with a significant difference in AMR gene composition across species (*P* = 0.001). The first cluster was characterized by a low AMR gene content, primarily limited to core resistance genes, and included *E. ludwigii* and *E. hormaechei* subsp. *oharae. E. kobei* clustered independently and harbored additional resistance genes belonging to the *APH*, *dfr*, *sul*, *mdtA*, and *bla*_NDM_ families. The third cluster exhibited a higher frequency and diversity of AMR genes, in addition to those observed in the second cluster. It included genes from the *bla*_OXA_, *bla*_CTX-M-15_, *bla*_TEM_, *AAC*, and *tet* families and was formed by *E. hormaechei* subsp. *hoffmannii*, *E. hormaechei* subsp. *xiangfangensis*, *E. hormaechei* subsp. *hormaechei*, and *E. hormaechei* subsp. *steigerwaltii*. The fourth cluster included *E. cloacae*, *E. roggenkampii*, *E. bugandensis*, *E. asburiae*, and the putative novel species. This group was characterized by a lower frequency of the AMR genes observed in the third cluster (Fig. 4b).

No relationship between genotypes of *mgrB*, *PhoPQ*, or *arnBCADTEF* and colistin resistance was observed when comparing sequences between colistin-resistant isolates and non-resistant isolates (data not shown).

### Virulome

**Fig 5:**
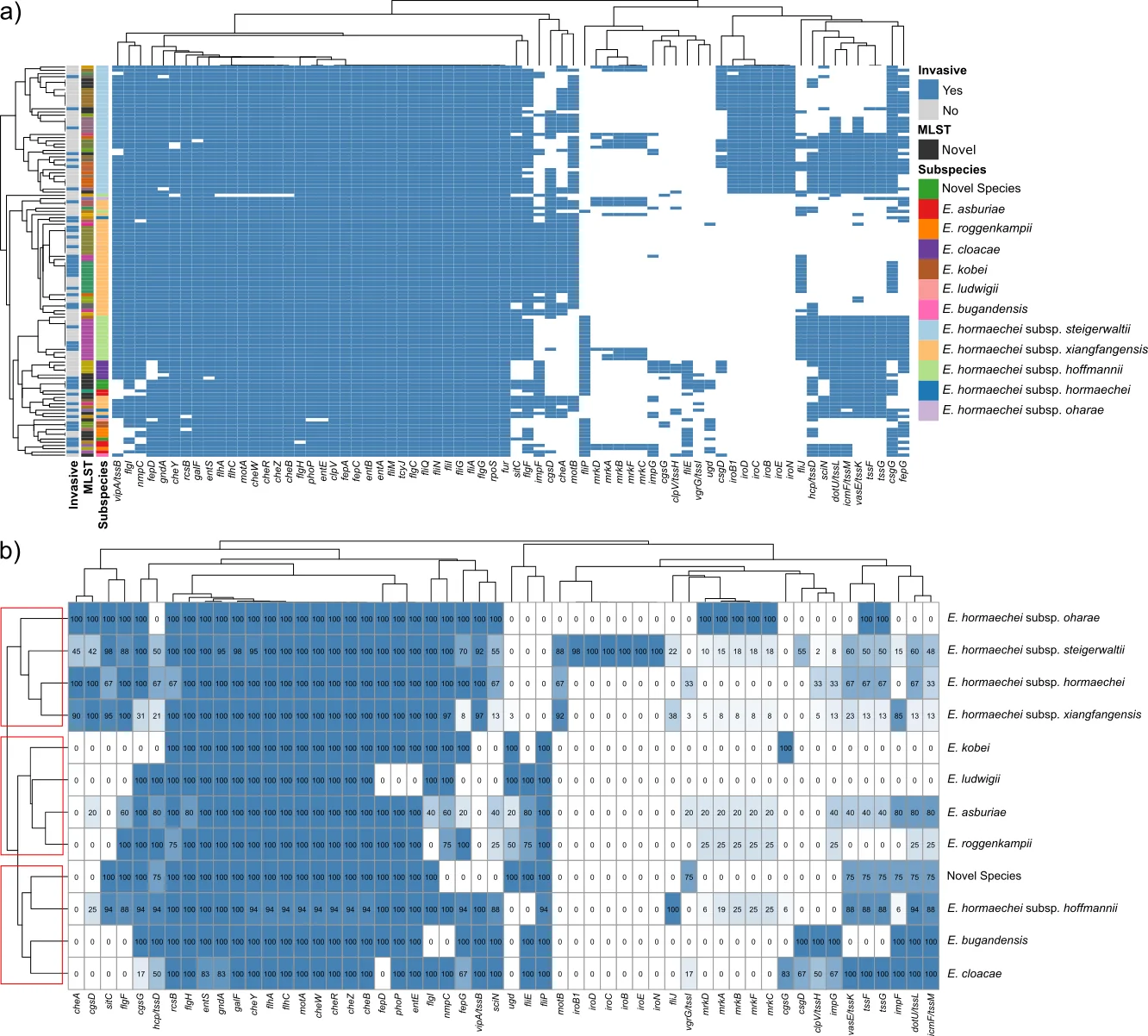
Virulome profiles of the sequenced isolates. (**a**) Distribution of virulence genes across all the included isolates. Only novel sequence types are included in the legend for visual purposes. (**b**) Frequency distribution of virulence genes by species and subspecies; numbers inside the boxes indicate the percentage of isolates of the corresponding species (rows) with the gene (columns). Genes with a frequency <2% (cloud genes) and >98% (core genes) are not shown for simplicity.

Isolates harbored an average of 51 VF genes, and the number of VF genes varied significantly across species (*P* < 0.01). Core VF genes were operons involved in chemotaxis (*cheBRWYZ*), flagella (*flgCGH*, *flhAC*, *fliAGIMNQ*, and *motA*), sulfur transport (*tcyJ*), iron acquisition (*entABES*, *fepAC*, and *fur*), O-antigen biosynthesis (*galF*, *gndA*, *rcsB*, and *rfb*), type VI secretion system (*clpV*), sigma factor regulation (*rpoS*), and the *PhoP* regulator (Fig. 5a).

Two VF-gene profiles were observed in *E. hormaechei* subsp. *steigerwaltii*: one characterized by a high frequency of iron-acquisition genes (*iroBDCEN* and *fepG*) coupled with additional T6SS genes (*TssDFGKLM*) and another devoid of these T6SS genes. *E. hormaechei* subsp. *hoffmannii* had a VF profile similar to the latter (mostly T6SS genes), which were more frequent in this subspecies (*P* < 0.005), while *E. hormaechei* subsp. *xiangfangensis* lacked T6SS and iron uptake genes (Fig. 5a).

The *iroBCDEN* gene cluster was only identified in *E. hormaechei* subsp. *steigerwaltii. flgG* was only detected in *E. ludwigii*, *fliF* in *E. roggenkampii*, and *csgE/flgN* in the putative novel species. *E. cloacae* exhibited a higher frequency in flagellar genes (*fliE* and *fliP*) and T6SS components (*clpV/tssH*, *tssF*, *tssG*, and *vasE/tssK*) (*P* < 0.05). Likewise, *E. asburiae* and the putative novel species showed significant enrichment of *fliE, fliP*, and *vgrG/tssI*, supporting their association with T6SS (*P* < 0.005). Moreover, although the number of VF genes was significantly lower in invasive isolates than in non-invasive isolates (*P* = 0.004), no specific VF genes were significantly associated with either invasive or non-invasive isolates (Fig. 5b).

### Mobilome

Plasmid sequences were identified in 89.3% (109/122) of the included isolates, encompassing 537 putative sequences: 220 were non-mobilizable, 196 were mobilizable, and 121 were conjugative. The most frequent replicon types were rep_cluster_2335 (*n* = 93), IncFIB/IncFII/rep_cluster_22 (*n* = 41), IncFIB/IncFII (*n* = 31), ColRNAl_rep_cluster_1987 (*n* = 30), IncFIB (*n* = 25), ColpVC (*n* = 12), IncFIA (*n* = 10), and IncFII (*n* = 10).

**Fig 6:**
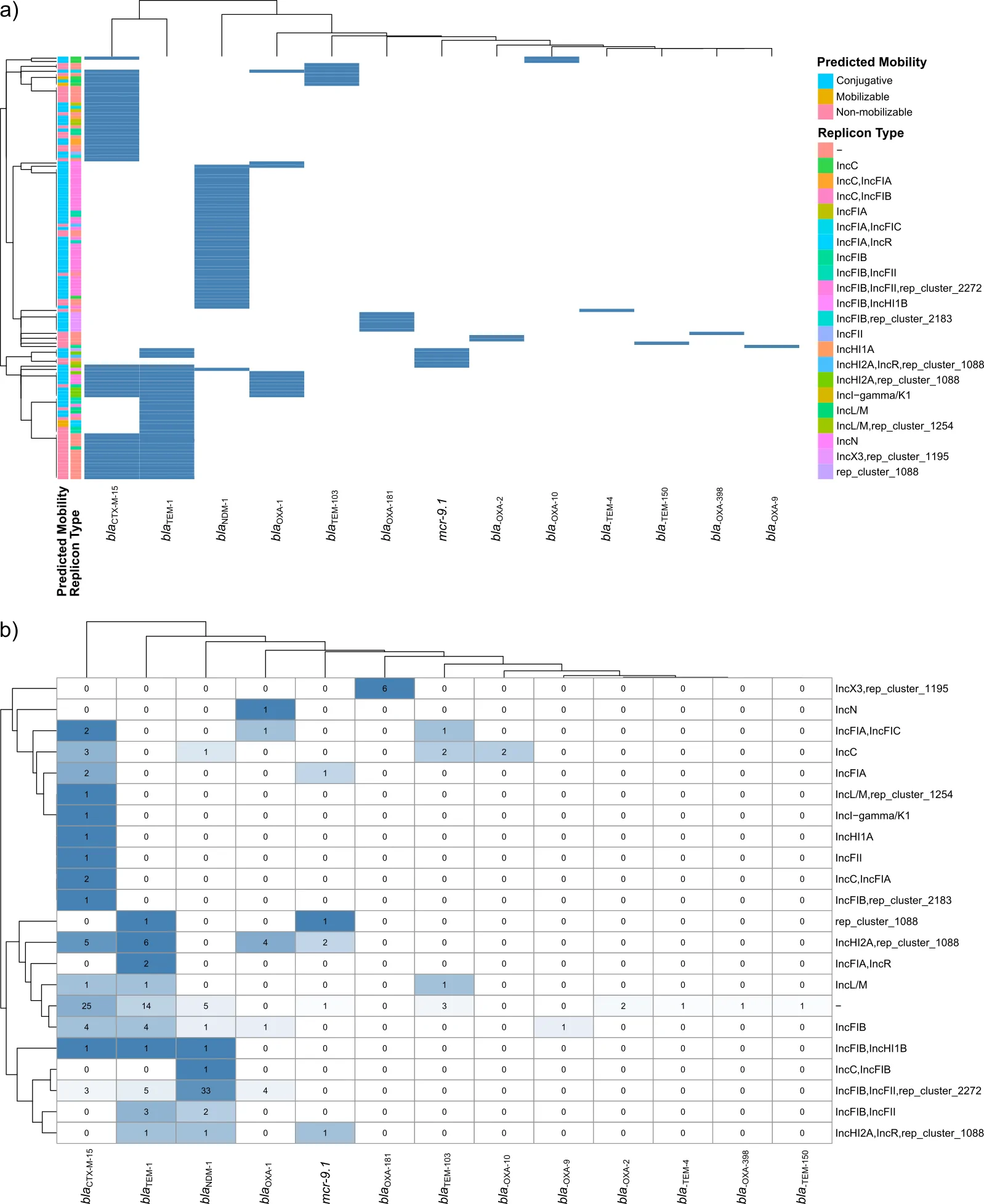
Distribution of antimicrobial resistance genes across plasmids. (**a**) β-lactamase genes detected across multiple plasmid replicon types. (**b**) Frequency matrix of antimicrobial resistance genes across plasmid replicon types, darker tones indicate a higher frequency relative to each row.

*bla*_CTX-M-15_ was the most frequent plasmid-associated ESBL (43.4% isolates), distributed among 14 replicon types, and identified 25 times in non-mobilizable plasmids (Table S6). Four distribution patterns were observed for *bla*_CTX-M-15_: co-localized with *bla*_TEM-1_, predominantly on non-mobilizable plasmids; (ii) co-localized with *bla*_TEM-1_and *bla*_OXA-1_, mostly on conjugative IncHIA/rep_cluster_1088 and IncFIB/IncFII plasmids; (iii) co-localized with *bla*_TEM-1_; and (iv) *bla*_CTX-M-15_ alone, found mostly in non-mobilizable plasmids from diverse replicon types (Fig. 6a). *bla*_OXA-181_ was exclusively detected in IncX3/rep_cluster_1196 plasmids, and *bla*_OXA-9_ was restricted to IncFIB plasmids. *bla*_OXA-2_, *bla*_TEM-4_, *bla*_TEM-150_, and *bla*_OXA-398_ were identified only in non-mobilizable plasmids (Fig. 6b).

**Fig 7:**
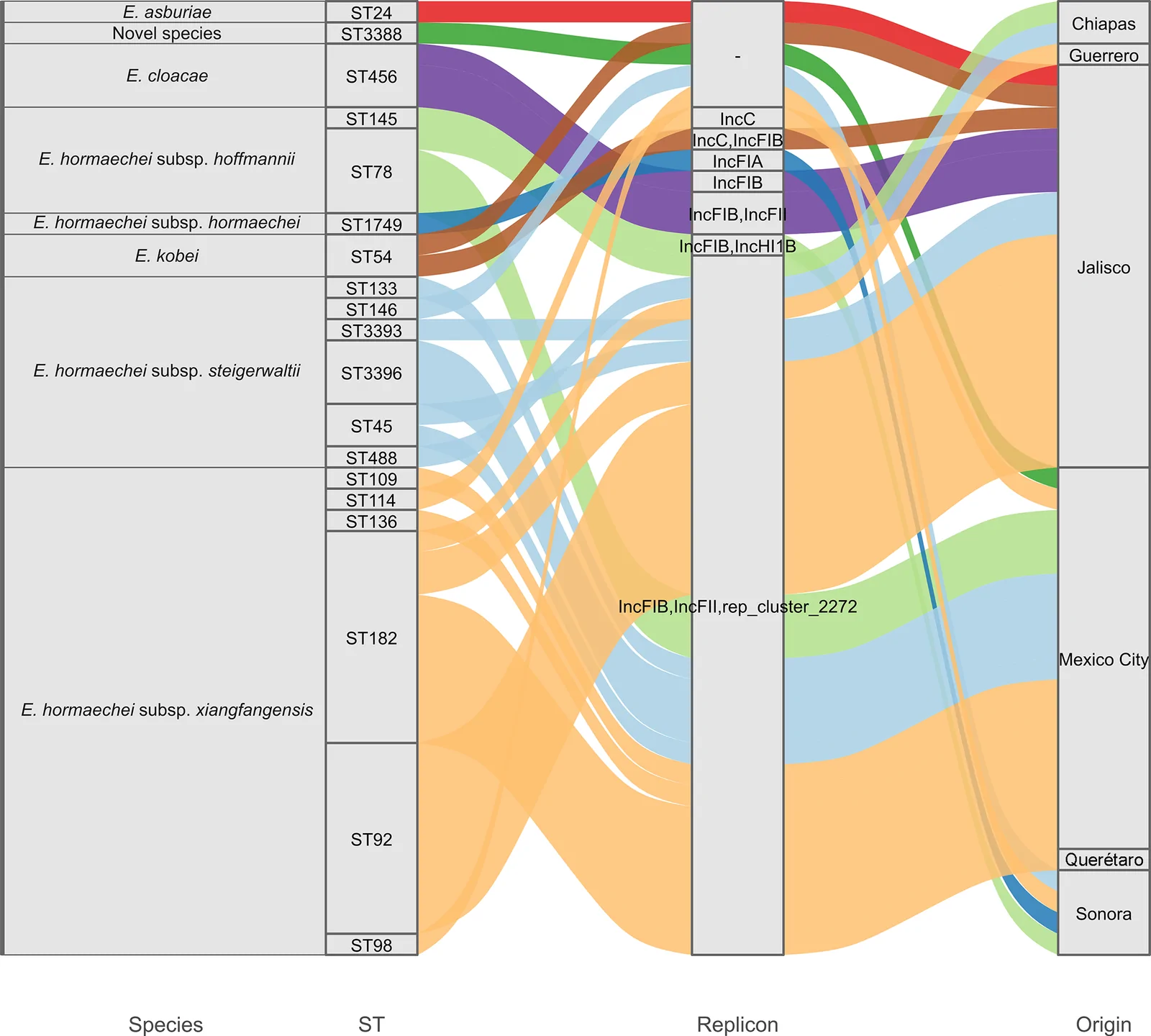
Relationship of *bla*_NDM-1_ plasmids across species, sequence types, and federal entities.

Of 48 isolates carrying *bla*_NDM-1_ according to whole-genome sequencing data, plasmid localization was observed in 45 isolates across five replicon types. These were detected in *E. hormaechei* subsp. *xiangfangensis*, *E. hormaechei* subsp. *steigerwaltii*, *E. hormaechei* subsp. *hoffmannii*, *E. hormaechei* subsp. *hormaechei*, *E. asburiae*, *E. kobei*, *E. chuandaensis*, and *E. cloacae*. The most frequent replicon type was the IncFIB/IncFII/rep_cluster_2272 (*n* = 37). These plasmids carried *bla*_NDM-1_ alone and were significantly associated with *E. hormaechei* subsp. *xiangfangensis* ST182 isolates (10/11) and ST92 isolates (9/9) (*P* < 0.01). *E. hormaechei* subsp. *xiangfangensis* ST182-IncFIB/IncFII/rep_cluster_2272 isolates were dispersed across four cities in central Mexico, while ST92 isolates were limited to a single healthcare center in Jalisco State (Fig. 7).

Synteny analysis revealed that these plasmids share a conserved backbone with a similar organization (Fig. S5). A comparison of this plasmid with the NCBI database identified the *Klebsiella pneumoniae* plasmid pCRE14 (CP074530.1) as the most similar plasmid. Three isolates were positive for *bla*_NDM-1_ but were not detected to be of plasmid origin; two were identified as *E. hormaechei* subsp. *steigerwaltii* ST45 and one as *E. cloacae* ST456. To determine if *bla*_NDM-1_ was integrated into the chromosome, the genomic context was analyzed. In all cases, *bla*_NDM-1_ was flanked by miniature inverted repeats. Contigs of these isolates, which contain *bla*_NDM-1_, were uploaded to NCBI BLAST, and the highest-scoring alignment was the previously mentioned pCRE14 plasmid.

### Phylogenetic analysis including public genomes

**Fig 8:**
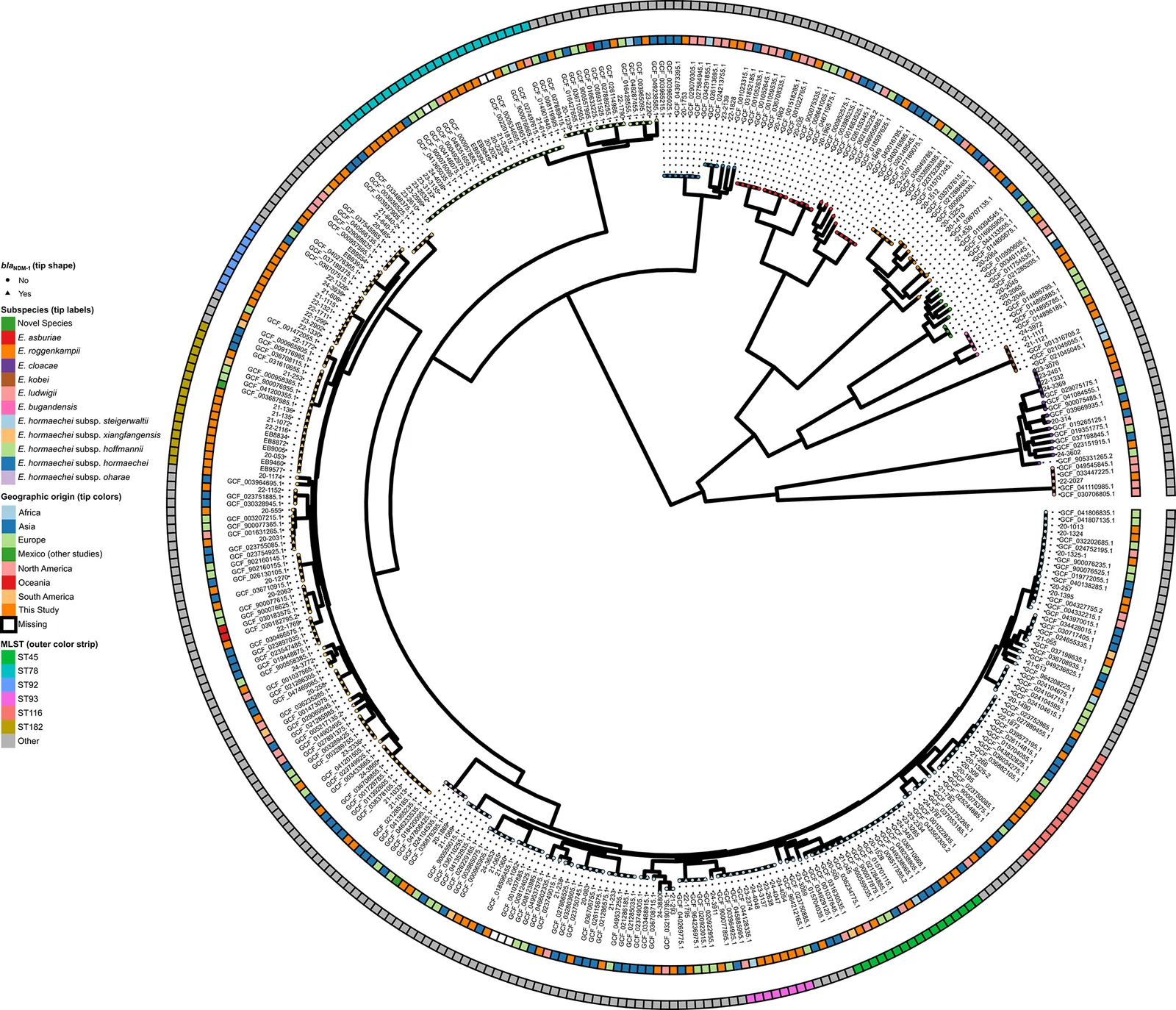
Core genome-based maximum-likelihood phylogenetic tree of the sequenced isolates and similar genomes from RefSeq.

Assemblies were compared to 7,909 public ECC genomes, and core alignment ML phylogenies were constructed on the selected genomes. The *E. hormaechei* subsp. *steigerwaltii* branch comprised 126 genomes, including the 40 newly sequenced isolates. Among these, 40 are of Asian, 25 of European, and 11 of North American origin, while genomes from Asia, Africa, and South America are represented at lower frequencies. The sequenced isolates from this study are evenly distributed across multiple subclades within the branch (Fig. 8).

The branch corresponding to *E. hormaechei* subsp. *xiangfangensis* contains 101 genomes. Of these, nine isolates are of North American origin, and most of the others originate from Asia (*n* = 32). The sequenced isolates from this study are primarily located within two major sub-branches: ST182 isolates cluster within a large branch comprising genomes from Mexico, South America, and Europe, while ST92 isolates form a distinct clade (Fig. 8).

Sequenced isolates of *E. hormaechei* subsp. *hoffmannii* are grouped within an ST78 branch, predominantly composed of European genomes. The remaining isolates are distributed across individual branches (Fig. 8).

Among the three *E. hormaechei* subsp. *hormaechei* isolates, two group exclusively with North American genomes in separate branches, while the remaining isolate clusters within a branch that includes genomes from Europe, Africa, and North America. *E. hormaechei* subsp. *oharae* grouped with minimal divergence with four isolates, one of which was isolated in Mexico, while the remaining genomes are from transcontinental sources (Europe/Asia).

The five *E. asburiae* isolates sequenced in this study fall into two principal lineages: two align with a predominantly Asian branch, while the other three cluster with strains from Europe and America. Among the six *E. cloacae* genomes, two group with Asian isolates, whereas the remaining four cluster within a separate clade that includes a genome from North America. The two related *E. kobei* isolates cluster exclusively with African strains. The only *E. ludwigii* isolate is related to North American sequences.

Among the four putative novel species isolates, three form independent branches, each most closely related to an Asian genome, while the fourth is similar to North American isolates. *E. roggenkampii* isolates are evenly distributed among genomes from Europe, North America, and Asia. The only *E. bugandensis* isolate clusters exclusively with genomes from Europe.

## DISCUSSION

Precise species-level identification within the ECC is critical due to differences in pathogenicity and AMR among its members (3). In our study, *E. hormaechei* was the predominant species, with the subspecies *E. hormaechei* subsp. *steigerwaltii*, *E. hormaechei* subsp. *xiangfangensis,* and *E. hormaechei* subsp. *hoffmannii* being the most frequently detected. Other ECC taxa were less common. This distribution is consistent with reports from Asia and Europe (3, 5, 9, 10, 40, 41).

The approach used in this study for taxonomic assignment led to the identification of a putative novel species phylogenetically related to *E. bugandensis*, *E. nematophilus*, and *E. chuandaensis*. In addition, our analyses suggest that *E. hormaechei* subsp. *hoffmannii* and *E. intestinihominis* likely represent the same species. These taxa exhibit ~99% ANI and cluster together in phylogenomic analyses. *E. intestinihominis* was initially described as a novel species in a gut microbiome study that compared newly sequenced isolates to publicly available genomes, without including other *Enterobacter* type strains (39). Based on the present evidence, *E. intestinihominis* should be considered *E. hormaechei* subsp. *hoffmannii*, rather than a distinct species.

While ANI is a robust and widely used metric for species assignment, taxa with low genomic divergence may deviate from the conventional threshold of 95%–96%, as observed within the genus *Enterobacter* (42). In such cases, core-genome and whole-genome phylogenomic analyses provide the most reliable approaches for accurate species and subspecies-level identification (43, 44).

*E. hormaechei* subsp. *steigerwaltii* and *E. hormaechei* subsp. *xiangfangensis* predominate in Asian studies (3, 10), whereas subspecies *E. hormaechei* subsp. *hoffmannii* is more frequently isolated in Europe (40, 41). Our data align more closely with the Asian pattern, though *E. hormaechei* subsp. *hoffmannii* was the third most common subspecies, reinforcing the clinical relevance of these three lineages within the ECC.

Resistance to broad-spectrum β-lactams exceeded 50% in our clinical isolates; this percentage is higher when compared to reports from Asia (3, 9). However, our call in the INVIFAR network for surveillance was for MDR organisms, which might introduce bias. This phenotype could be related to the high prevalence of genes encoding ESBLs, particularly *bla*_CTX-M-15_ (48% of isolates), one of the most described ESBLs in *Enterobacter* (2). Carbapenem resistance was detected in 45% of the included isolates, over fourfold higher than the rates reported in large-scale surveillance studies (~10%) (3, 9, 45), and this resistance was associated with the presence of the *bla*_NDM-1_ gene. Most reports from Asia identify *bla*_NDM,_ whereas *bla*_KPC_ families predominate in studies from America, raising the possibility of transcontinental spread, though this assumption is limited by the fact that no recent large-scale studies have been conducted in recent years in America (11). Supporting this interpretation, our phylogenetic analysis with public genomes showed a low proportion of similar genomes from North America. Instead, most were from Asian and European origins.

Colistin resistance in our study was detected in 10% of clinical isolates, contrasting with other studies reporting 20% (46) to 70% (3, 9, 45) of resistant isolates. Only one colistin-resistant isolate carried *mcr*, and eight *mcr*-positive isolates were non-resistant to colistin. Consistent with our results, Bitar et al. (47) reported the detection of *mcr* in *Enterobacterales*, including four isolates belonging to the *Enterobacter cloacae* complex, none of which exhibited phenotypic resistance to colistin. Resistance to colistin in our collection of clinical isolates might arise through mechanisms independent of *mcr* that were not explored in this study, such as mutations in the *arn* operon or its regulators *phoPQ* and *mgrB,* which lead to modifications of lipid A (48). Of note is that all the isolates from the putative novel species were resistant to colistin by an undefined mechanism.

Although *E. hormaechei* subspecies exhibited higher frequency of isolation, AMR rates, and *bla*_NDM-1_ frequency, colistin resistance was observed in only one isolate. In contrast, colistin resistance was detected among isolates of *E. cloacae*, *E. asburiae*, *E. roggenkampii*, *E. kobei,* and the putative novel species. These patterns could reflect clonal dissemination of colistin-resistant isolates, particularly *E. cloacae* ST456 and the putative novel species, which exhibit strong phylogenetic relatedness.

*E. hormaechei* subsp. *steigerwaltii* was represented by 16 distinct STs and 5 novel STs, with ST93, ST45, and ST116 being the most frequent. ST93 isolates originated exclusively from two healthcare centers in Sonora (North Mexico). ST93 is a globally widespread clone (11) commonly associated with *bla*_NDM-1_ (10, 45). However, in our study, clinical isolates corresponding to this ST were susceptible to carbapenems, and only *bla*_OXA-like_ enzymes were identified. ST45 has been reported as a *bla*_KPC_ carrier in Colombia (49), Chile (50), and Argentina (51), while it remains largely underreported in Asia/Europe. In the present study, ST45 was dispersed across five different cities in Mexico. On the contrary, ST116 isolates lacking carbapenemases were dispersed across four different cities of Mexico and have been mostly reported in studies from Asia (52, 53). The limited confinement of ST92 to the state of Sonora (Northern Mexico) might suggest an outbreak of this clone.

The most frequent STs for *E. hormaechei* subsp. *xiangfangensis* were ST182 and comprised 11 isolates distributed across six attention centers in different cities of central Mexico.

ST182 is recognized as an epidemiologically emerging lineage, commonly associated with *bla*_NDM_-harboring plasmids with the IncFIB/IncFII/rep_cluster_2272 replicon type (54, 55), as was detected in this study. The association of ST182 with this replicon type has been described in Europe (56, 57). A 2015 report from Mexico described the presence of *bla*_NDM-1_ in a ST182 isolate; however, the gene was carried on an IncFII plasmid (58). Phylogenetic analysis shows a strong relationship between all the ST182 isolates detected in this study, even when these were isolated from distant geographic locations. Additionally, comparison with public genomic data showed a close relatedness to a Mexican isolate and South American isolates.

The second most frequent ST of *E. hormaechei* subsp. *xiangfangensis* was ST92, which was limited to a single healthcare center in Central Mexico (Jalisco) from 2021 to 2024. These isolates showed a near-0 distance in the phylogenetic analysis and harbored the *bla*_NDM-1_ IncFIB/IncFII/rep_cluster_2272 plasmid. Few reports have documented the appearance of this ST and have mostly associated it with *bla*_VIM_ (59, 60), contrasting with our results. Furthermore, phylogenetic analysis with RefSeq genomes showed that these strains form an independent clade, suggesting a potential outbreak of ST92 localized in a single attention center/city.

A clonal distribution was observed for *E. hormaechei* subsp. *hoffmannii*, with 13 ST78 identified across Northern, Central, and Southern Mexican cities between 2020 and 2024. These isolates originated from eight different attention centers, and some of the strains harbored the IncFIB/IncFII/rep_cluster_2272 *bla*_NMD-1_ plasmid. ST78 is recognized as a high-risk, ESBL-producing international clone reported from a 2008–2014 collection study (8), and since then, it has been reported in Europe (61) and America (62). This is consistent with phylogenetic analysis with public genomes, where the ST78 cluster includes genomes from multiple countries. For *E. cloacae*, ST456 was the most frequent ST, represented by four isolates detected in two attention centers from the same city. Three of these isolates were resistant to colistin and lacked the *mcr* gene (49, 63). This ST has been reported in a few studies and has not been associated with colistin resistance, contrary to our results. The remaining isolates comprised unique or novel STs, with a diversity of STs observed, including novel STs.

AMR gene content analysis identified two primary clusters: one characterized by an elevated frequency of AMR determinants. *E. hormaechei* subspecies *hoffmannii*, *xiangfangensis*, and *steigerwaltii* belonged to this high-AMR group, which may explain their frequent isolation and underscores their potential to acquire AMR determinants. In contrast, analysis of VF gene content revealed only subtle differences among isolates, with the most notable distinction being the presence of iron-acquisition genes in *E. hormaechei* subsp. *steigerwaltii*. This observation suggests that the VF genes identified here could represent baseline virulence determinants essential for survival in clinical environments, as species with lower isolation frequencies exhibited similar VF profiles to those isolated more frequently.

The IncFIB/IncFII/rep_cluster_2272 plasmid was the principal mobile element associated with the presence of the *bla*_NDM-1_ gene. A comprehensive analysis of isolates carrying this plasmid uncovered that it has a conserved structure and is mainly located in five cities of central Mexico and one of northern Mexico, across eight different healthcare centers. Its conjugative nature is highlighted by the fact that it was present in five different ECC species.

While short-read sequencing is not the gold standard for plasmid reconstruction, MOB-suite is designed to mitigate limitations of short-read data and shows high sensitivity (≈95%) for plasmids, although it has limited ability to detect novel plasmids. Long-read sequencing is recommended when complete plasmid assemblies are required (35). In this study, plasmids were reconstructed using an 80% coverage threshold and validated by BLAST comparisons against related reference plasmids, yielding high coverage values (Fig. S5).

This study has limitations. First, extended mechanisms for colistin resistance beyond the presence of the *mcr* gene, such as phenotypic evidence of LPS modifications, were not explored. Second, phenotypic tests for VF analysis were not performed, and therefore, interpretations regarding the clinical relevance of the virulome should be made with caution. Third, genomes from *E. hormaechei* subspecies were overrepresented in our data set, reflecting the natural distribution of ECC species in the sampled population. Finally, plasmid analysis is limited by short-read sequencing, which restricts full plasmid reconstructions (64), even though we leveraged the unmapped-read assembly approach to reconstruct these sequences.

### Conclusion

Among the included isolates, *E. hormaechei* was the most frequent species identified, with *E. hormaechei* subsp. *xiangfangensis*, *E. hormaechei* subsp. *steigerwaltii*, and *E. hormaechei* subsp. *hoffmannii* as the predominant subspecies. *E. hormaechei* subsp. *xiangfangensis* showed higher overall resistance rates. Phylogenetic analysis and distribution of *bla*_NDM-1_ suggest transcontinental dissemination. Phylogenetic analysis and MLST revealed both widespread (ST182) and localized (ST92) sequence types within *E. hormaechei* subsp. *xiangfangensis*. Similarly, *E. hormaechei* subsp. *steigerwaltii* included widespread (ST45 and ST116) and localized (ST93) clones; *E. hormaechei* subsp. *hoffmannii* was represented by the widespread high-risk ST78 clone. The main ESBL and carbapenemase genes detected were *bla*_CTX-M-15_ and *bla*_NDM-1_, both of which were largely plasmid-associated, with the latter linked to IncFIB/IncFII/rep_cluster_2272 replicon types. Based on genomic evidence, we propose *E. intestinihominis* as a synonym of *E. hormaechei* subsp. *hoffmannii*. Finally, we describe a putative novel species within the *Enterobacter* genus.

## Data Availability

Assemblies were uploaded to NCBI under project number PRJNA1297765.

## SUPPLEMENTAL MATERIAL

Figure S1 spectrum.04019-25-s0001.pdf Average nucleotide identity (ANI) of the sequenced isolates and reference genomes of *Enterobacter* spp.

Supplemental figures spectrum.04019-25-s0002.docx Fig. S2 to S5.

Supplemental tables spectrum.04019-25-s0003.docx Tables S1 to S6.

ASM does not own the copyrights to Supplemental Material that may be linked to, or accessed through, an article. The authors have granted ASM a non-exclusive, world-wide license to publish the Supplemental Material files. Please contact the corresponding author directly for reuse.
