# Supplementary material for: Genomic characterization of Enterobacter isolates highlights widespread ST78 high-risk clone and plasmid-mediated dissemination of blaNDM-1
Source: Microbiol Spectr. 2026 Jun 15;14(7):e04019-25. doi: 10.1128/spectrum.04019-25 (PMC13339806; doi:10.1128/spectrum.04019-25)

Figure S1. Average nucleotide identity (ANI) of the sequenced isolates and reference genomes of *Enterobacter* spp.

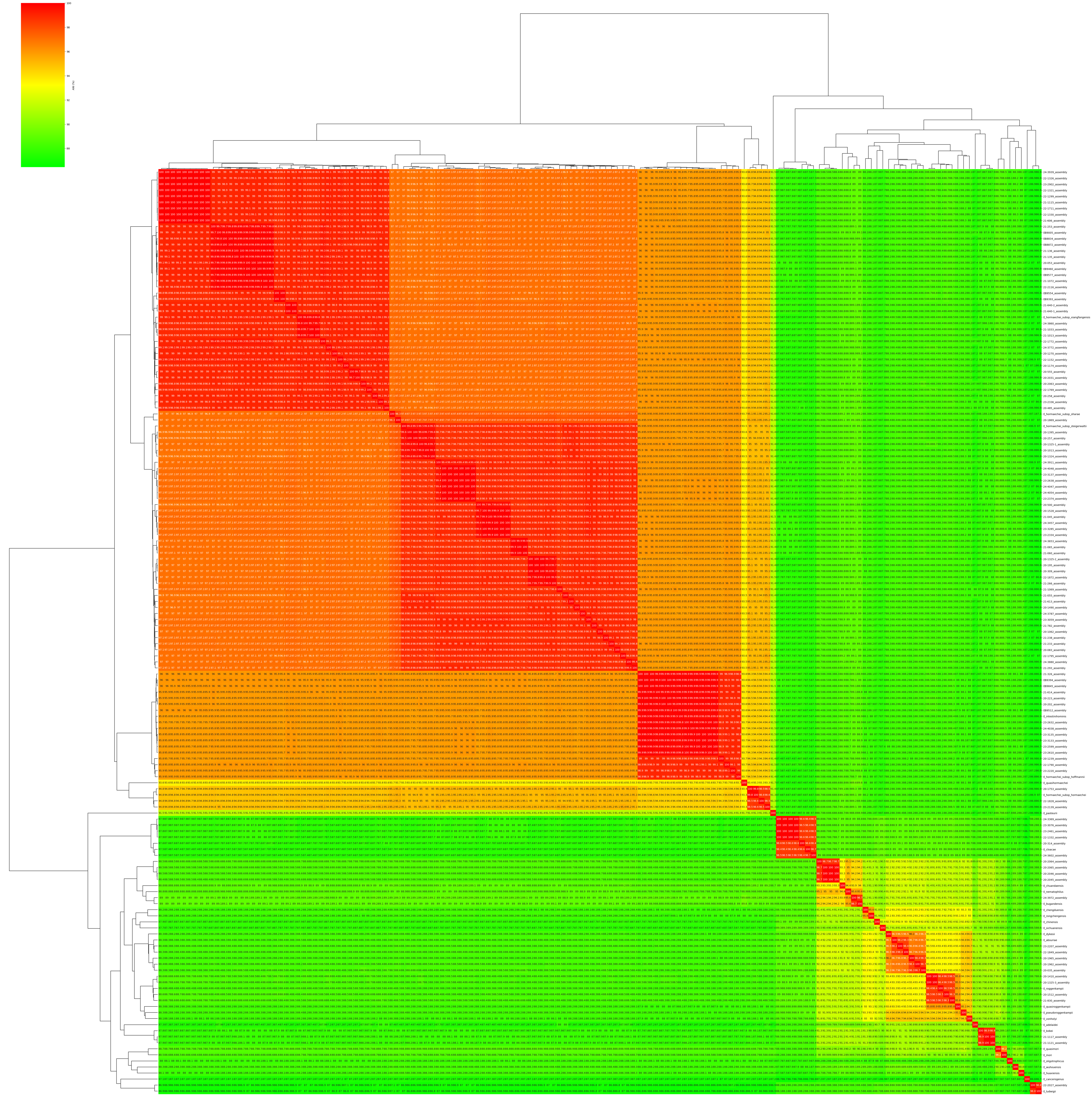

Supplement: Figure S1 — Average nucleotide identity (ANI) of the sequenced isolates and reference genomes of Enterobacter spp. [file spectrum.04019-25-s0001.pdf]
