# Supplementary material for: Genomic characterization of Enterobacter isolates highlights widespread ST78 high-risk clone and plasmid-mediated dissemination of blaNDM-1
Source: Microbiol Spectr. 2026 Jun 15;14(7):e04019-25. doi: 10.1128/spectrum.04019-25 (PMC13339806; doi:10.1128/spectrum.04019-25)
Supplement: Supplemental figures — Fig. S2 to S5. [file spectrum.04019-25-s0002.docx]

**Figure S2.** Results from the Type Strain Genome Server for isolates with inconclusive taxonomy. The panel on the right summarizes TYGS-derived clustering and genome metrics for each isolate


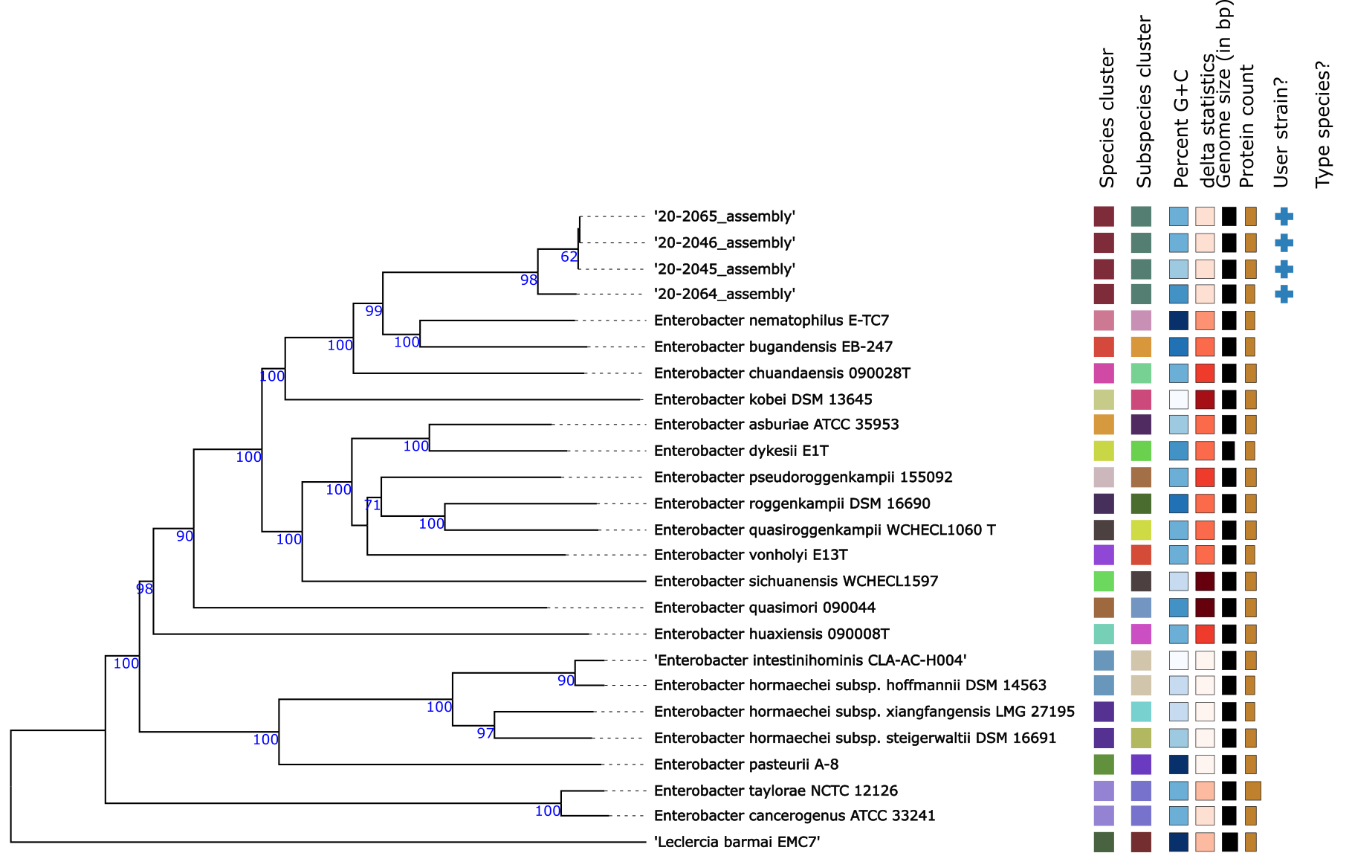


**Figure S3.** Results from the Type Strain Genome Server for isolates identified as *E. hormaechei* subsp. *hoffmanni*. The panel on the right summarizes TYGS-derived clustering and genome metrics for each isolate


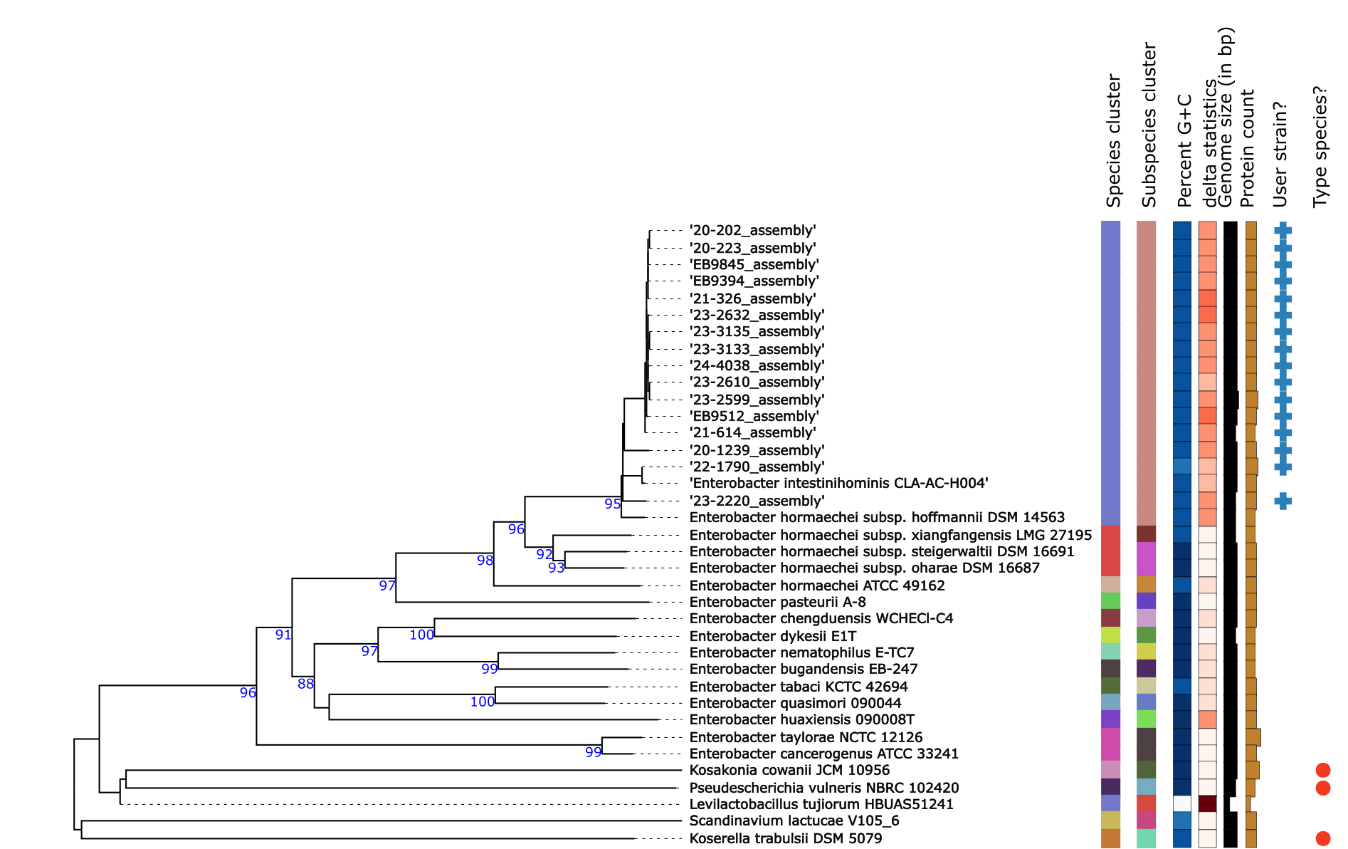


**Figure S4.** goeBURST analysis of the identified sequence types (STs). Nodes represent each ST, while edge labels (red) indicate the number of allelic differences between multi-*locus* sequence type genes.


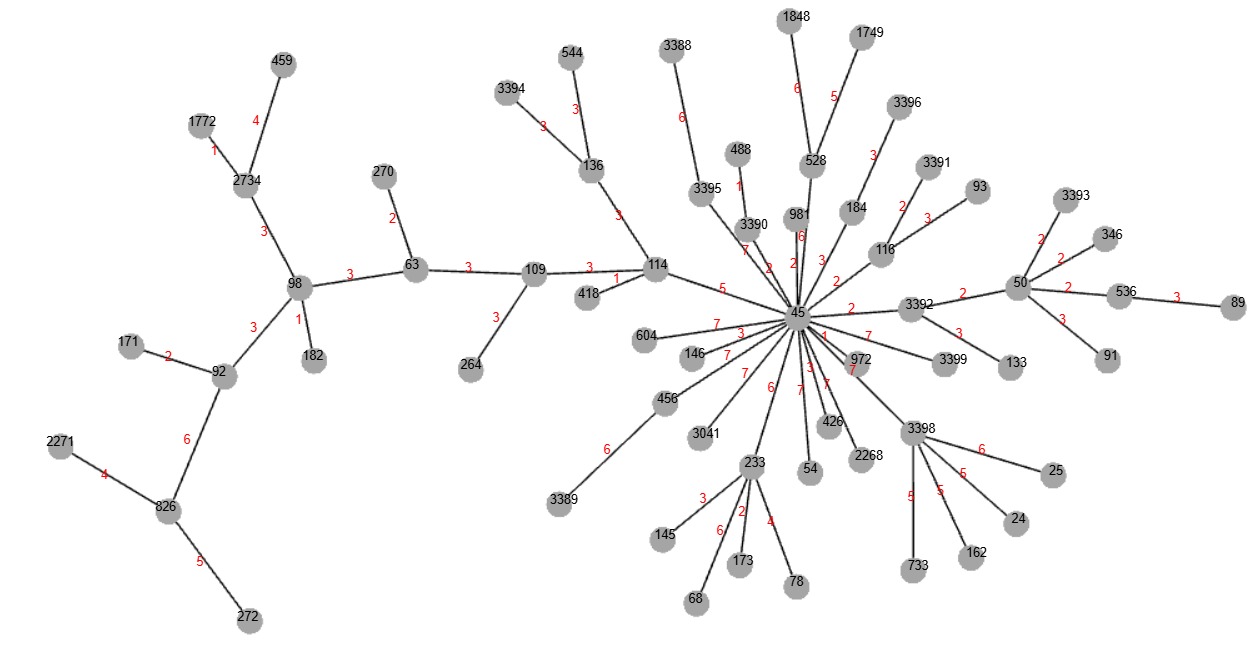


**Figure S5.** Annotation and synteny of representative IncFIB/IncFII/rep_cluster_2272 plasmids harboring *bla*_NDM-1_.

**
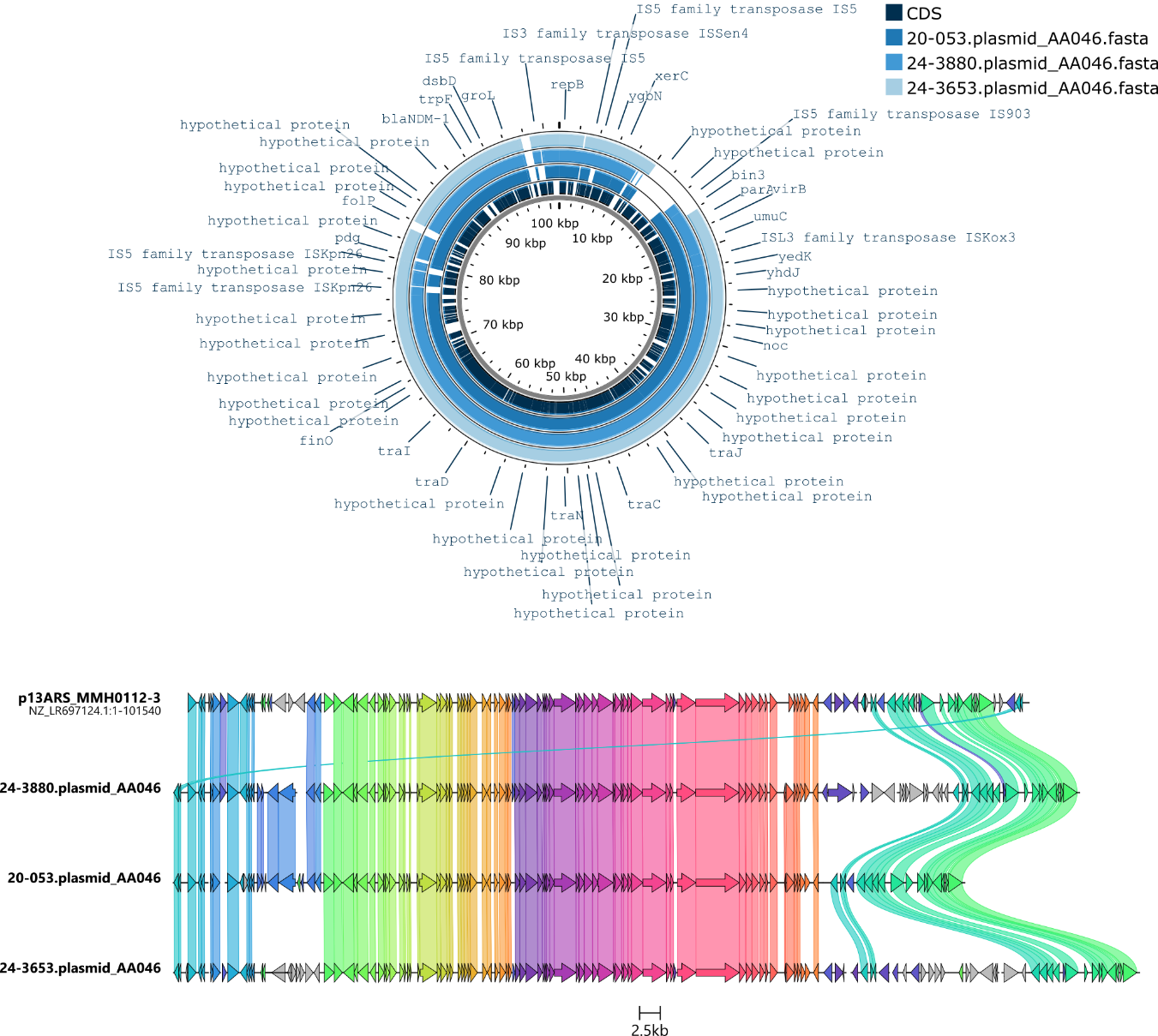
**
