# Supplementary material for: Genomic characterization of Enterobacter isolates highlights widespread ST78 high-risk clone and plasmid-mediated dissemination of blaNDM-1
Source: Microbiol Spectr. 2026 Jun 15;14(7):e04019-25. doi: 10.1128/spectrum.04019-25 (PMC13339806; doi:10.1128/spectrum.04019-25)
Supplement: Supplemental tables — Tables S1 to S6. [file spectrum.04019-25-s0003.docx]

**Supplementary Table 1. Distribution and characteristics of participating centers**

| **Center** | **State** | **Pu/Pr** | **Hospital type** | **Level attention**  **(1st, 2nd, 3rd)** | **Num. total beds** | **Num ICU beds** |
| --- | --- | --- | --- | --- | --- | --- |
| Laboratorio de Microbiología Hospital Civil de Guadalajara “Fray Antonio Alcalde” | Jalisco | Pu | Spe | 3rd | 830 | 100 |
| Hospital General de León | Guanajuato | Pu | Gen | 2nd | 531 | 18 |
| Hospital General “Dr Agustin O´Horán” | Yucatán | Pu | Spe | 2nd | 475 | 11 |
| Hospital General del Estado de Sonora | Sonora | Pu | Spe | 3rd | 305 | 10 |
| Laboratorio de Microbiología Clínica, Instituto Nacional de Rehabilitación | Mexico City | Pu | Spe | 3rd | 245 | 18 |
| Instituto Nacional de Cardiología “Ignacio Chávez” | Mexico City | Pu | Spe | 3rd | 208 | 30 |
| Hospital Regional Alta Especialidad ISSSTE Monterrey | Nuevo León | Pu | Spe | 3rd | 200 | 10 |
| Hospital Regional de Alta Especialidad Bicentenario de la Independencia ISSSTE | Mexico State | Pu | Spe | 3rd | 196 | 7 |
| Hospital Regional de Alta Especialidad del Bajío | Guanajuato | Pu | Spe | 3rd | 184 | 16 |
| Hospital General de Zona Nueva Frontera | Chiapas | Pu | Gen | 2nd | 180 | 28 |
| Laboratorio de Microbiología, Instituto Nacional de Cancerología | Mexico City | Pu | Spe | 3rd | 165 | 6 |
| Centro Médico “Doctor Ignacio Chávez” ISSSTESON | Sonora | Pu | Gen | 2nd | 164 | 4 |
| Instituto Nacional de Enfermedades Respiratorias "Isamael Cosio Villegas" | Mexico City | Pu | Spe | 3rd | 161 | 15 |
| Centenario Hospital “Miguel Hidalgo” | Aguascalientes | Pu | Spe | 3rd | 144 | 35 |
| Hospital Regional de Alta Especialidad de Oaxaca | Oaxaca | Pu | Spe | 3rd | 66 | 10 |
| Hospital de Niño “Dr Federico Gómez Santos” | Coahuila de Zaragoza | Pu | Ped | 3rd | 44 | 13 |
| Hospital de la Madre y el Niño Guerrerense | Guerrero | Pu | M&Ch | 3rd | 32 | 3 |
| Departamento de Microbiología, Hospital la Luz Morelia | Michoacán | Pu | Gen | 2nd | 28 | 3 |
| Hospital General de Zona No. 21 IMSS Jalisco | Jalisco | Pu | Gen | 2nd | 139 | 9 |
| Hospital de Alta Especialidad de Veracruz | Veracruz | Pu | Spe | 3rd | 240 | 5 |
| Hospital HMAS Queretaro by Hospitales MAC | Querétaro | Pr | Spe | 3rd | 87 | No data |
| **Laboratory outside healthcare facilities** |  |  |  |  |  |  |
| Laboratorio de Microbiología Clínica, Universidad Autónoma de Guerrero | Guerrero | Pr | NA | NA | NA | NA |
| Laboratorio DIPROMI | Michoacán | Pr | NA | NA | NA | NA |
| Diagnóstico Médico Integral Pasteur | Mexico State | Pr | NA | NA | NA | NA |

Abbreviations: Pr: Private, Pu: Public, NA: Not apply, Gen: General, Ped: Pediatric, Spe: Specialties, M&Ch: Mother and Child, ICU: intensive care unit.

**Supplementary Table 2. Isolate metadata and ribosomal MLST (rMLST) assignment results.**

| Isolate Name | Isolation year | MLST | Federal entity | Source | Invasive | rMLST assignment | rMLST confidence (%) | |
| --- | --- | --- | --- | --- | --- | --- | --- | --- |
| ***E. hormaechei* subsp. *steigerwaltii*** | | | | | | | |  |
| 20-083 | 2020 | 346 | Michoacan | Urine | No | *E. hormaechei* | 100 | |
| 20-1013 | 2020 | 91 | Sonora | Urine | No | *E. hormaechei* | 100 | |
| 20-1062 | 2020 | 604 | Michoacan | Wound | No | *E. hormaechei* | 100 | |
| 20-1324 | 2020 | 91 | Oaxaca | Urine | No | *E. hormaechei* | 100 | |
| 20-1325 | 2020 | 91 | Oaxaca | Urine | No | *E. hormaechei* | 100 | |
| 20-1325-2 | 2020 | 116 | Oaxaca | Urine | No | *E. hormaechei* | 100 | |
| 20-1395 | 2020 | 426 | Jalisco | Respiratory | No | *E. hormaechei* | 100 | |
| 20-1490 | 2020 | 89 | Mexico City | Wound | No | *E. hormaechei* | 100 | |
| 20-1528 | 2020 | 45 | Guanajuato | Blood | Yes | *E. hormaechei* | 100 | |
| 20-195 | 2020 | 116 | Aguascalientes | Respiratory | No | *E. hormaechei* | 100 | |
| 20-257 | 2020 | 426 | Jalisco | Blood | Yes | *E. hormaechei* | 100 | |
| 20-309 | 2020 | 116 | Mexico City | Urine | No | *E. hormaechei* | 100 | |
| 20-500 | 2020 | 45 | Veracruz | Blood | Yes | *E. hormaechei* | 100 | |
| 21-045 | 2021 | 45 | Jalisco | Secretion | No | *E. hormaechei* | 100 | |
| 21-055 | 2021 | 184 | Mexico City | Urine | No | *E. hormaechei* | 100 | |
| 21-060 | 2021 | 3396 | Mexico City | Urine | No | *E. hormaechei* | 100 | |
| 21-065 | 2021 | 3396 | Mexico City | Urine | No | *E. hormaechei* | 100 | |
| 21-1069 | 2021 | 133 | Mexico City | Secretion | No | *E. hormaechei* | 100 | |
| 21-233 | 2021 | 981 | Guanajuato | Secretion | No | *E. hormaechei* | 100 | |
| 21-238 | 2021 | 536 | Guanajuato | Respiratory | No | *E. hormaechei* | 100 | |
| 21-266 | 2021 | 116 | Yucatan | Wound | No | *E. hormaechei* | 100 | |
| 21-293 | 2021 | 3390 | Mexico City | Abscess | Yes | *E. hormaechei* | 100 | |
| 21-613 | 2021 | 3391 | Oaxaca | Respiratory | No | *E. hormaechei* | 100 | |
| 21-782 | 2021 | 3392 | Guerrero | Wound | No | *E. hormaechei* | 100 | |
| 22-1795 | 2022 | 146 | Sonora | Feces | No | *E. hormaechei* | 100 | |
| 22-1872 | 2022 | 116 | Mexico City | Respiratory | No | *E. hormaechei* | 100 | |
| 23-2334 | 2023 | 45 | Mexico City | Urine | No | *E. hormaechei* | 100 | |
| 23-2374 | 2023 | 93 | Sonora | Urine | No | *E. hormaechei* | 100 | |
| 23-2638 | 2023 | 93 | Sonora | Urine | No | *E. hormaechei* | 100 | |
| 23-3059 | 2023 | 972 | Mexico State | Wound | No | *E. hormaechei* | 100 | |
| 23-3137 | 2023 | 93 | Sonora | Urine | No | *E. hormaechei* | 100 | |
| 23-3285 | 2023 | 45 | Mexico City | Urine | No | *E. hormaechei* | 100 | |
| 24-3457 | 2024 | 45 | Mexico City | Urine | No | *E. hormaechei* | 100 | |
| 24-3653 | 2024 | 3396 | Mexico City | Respiratory | No | *E. hormaechei* | 100 | |
| 24-3787 | 2024 | 50 | Jalisco | Biopsy | Yes | *E. hormaechei* | 100 | |
| 24-3880 | 2024 | 488 | Chiapas | Blood | Yes | *E. hormaechei* | 100 | |
| 24-3911 | 2024 | 3393 | Jalisco | Biopsy | Yes | *E. hormaechei* | 100 | |
| 24-4047 | 2024 | 93 | Sonora | Urine | No | *E. hormaechei* | 100 | |
| 24-4048 | 2024 | 93 | Sonora | Urine | No | *E. hormaechei* | 100 | |
| 24-4054 | 2024 | 93 | Sonora | Urine | No | *E. hormaechei* | 100 | |
| ***E. hormaechei* subsp. *xiangfangesis*** | | | | | | | |  |
| 20-053 | 2020 | 182 | Queretaro | Blood | Yes | *E. hormaechei* | 100 | |
| 20-1174 | 2020 | 3394 | Mexico City | Urine | No | *E. hormaechei* | 100 | |
| 20-1270 | 2020 | 63 | Guerrero | Blood | Yes | *E. hormaechei* | 100 | |
| 20-2031 | 2020 | 264 | Mexico City | Biopsy | Yes | *E. hormaechei* | 100 | |
| 20-2063 | 2020 | 114 | Mexico City | Wound | No | *E. hormaechei* | 100 | |
| 20-258 | 2020 | 171 | Jalisco | Urine | No | *E. hormaechei* | 100 | |
| 20-485 | 2020 | 459 | Veracruo | Blood | Yes | *E. hormaechei* | 100 | |
| 20-555 | 2020 | 264 | Mexico City | Blood | Yes | *E. hormaechei* | 100 | |
| 21-1013 | 2021 | 544 | Jalisco | Blood | Yes | *E. hormaechei* | 100 | |
| 21-1033 | 2021 | 544 | Jalisco | Urine | No | *E. hormaechei* | 100 | |
| 21-1072 | 2021 | 182 | Mexico City | Urine | No | *E. hormaechei* | 100 | |
| 21-1115 | 2021 | 92 | Jalisco | Secretion | No | *E. hormaechei* | 100 | |
| 21-135 | 2021 | 182 | Jalisco | Urine | No | *E. hormaechei* | 100 | |
| 21-136 | 2021 | 182 | Jalisco | Catheter | No | *E. hormaechei* | 100 | |
| 21-253 | 2021 | 182 | Sonora | Wound | No | *E. hormaechei* | 100 | |
| 21-606 | 2021 | 92 | Jalisco | Catheter | No | *E. hormaechei* | 100 | |
| 21-640 | 2021 | 270 | Sonora | Urine | No | *E. hormaechei* | 100 | |
| 21-640-2 | 2021 | 270 | Sonora | Urine | No | *E. hormaechei* | 100 | |
| 22-1152 | 2022 | 2734 | Mexico City | Respiratory | No | *E. hormaechei* | 100 | |
| 22-1299 | 2022 | 92 | Jalisco | Blood | Yes | *E. hormaechei* | 100 | |
| 22-1321 | 2022 | 92 | Jalisco | Secretion | No | *E. hormaechei* | 100 | |
| 22-1326 | 2022 | 92 | Jalisco | Urine | No | *E. hormaechei* | 100 | |
| 22-1330 | 2022 | 92 | Jalisco | CSF | Yes | *E. hormaechei* | 100 | |
| 22-1711 | 2022 | 92 | Jalisco | Urine | No | *E. hormaechei* | 100 | |
| 22-1769 | 2022 | 418 | Sonora | Respiratory | No | *E. hormaechei* | 100 | |
| 22-1772 | 2022 | 98 | Sonora | Urine | No | *E. hormaechei* | 100 | |
| 22-2116 | 2022 | 182 | Guerrero | Urine | No | *E. hormaechei* | 100 | |
| 23-2336 | 2023 | 136 | Mexico City | Urine | No | *E. hormaechei* | 100 | |
| 23-2902 | 2023 | 92 | Jalisco | Blood | Yes | *E. hormaechei* | 100 | |
| 24-3772 | 2024 | 1772 | Mexico State | Urine | No | *E. hormaechei* | 100 | |
| 24-3860 | 2024 | 544 | Jalisco | Blood | Yes | *E. hormaechei* | 100 | |
| 24-3939 | 2024 | 92 | Jalisco | Blood | Yes | *E. hormaechei* | 100 | |
| EB8834 | 2024 | 182 | Mexico City | Biopsy | Yes | *E. hormaechei* | 100 | |
| EB8872 | 2024 | 182 | Mexico City | Drainage | Yes | *E. hormaechei* | 100 | |
| EB9005 | 2024 | 182 | Mexico City | Blood | Yes | *E. hormaechei* | 100 | |
| EB9393 | 2024 | 109 | Mexico City | Biopsy | Yes | *E. hormaechei* | 98 | |
| EB9460 | 2024 | 182 | Mexico City | Biopsy | Yes | *E. hormaechei* | 100 | |
| EB9554 | 2024 | 109 | Mexico City | Biopsy | Yes | *E. hormaechei* | 98 | |
| EB9577 | 2024 | 182 | Mexico City | Biopsy | Yes | *E. hormaechei* | 100 | |
| ***E. hormaechei* subsp. *hoffmannii*** | | | | | | | |  |
| 20-1239 | 2020 | 233 | Nuevo Leon | Urine | No | *E. hormaechei* | 100 | |
| 20-202 | 2020 | 78 | Aguascalientes | Blood | Yes | *E. hormaechei* | 100 | |
| 20-223 | 2020 | 78 | Jalisco | Blood | Yes | *E. hormaechei* | 100 | |
| 21-326 | 2021 | 78 | Chiapas | Urine | No | *E. hormaechei* | 100 | |
| 21-614 | 2021 | 78 | Oaxaca | Blood | Yes | *E. hormaechei* | 100 | |
| 22-1790 | 2022 | 145 | Sonora | Respiratory | No | *E. hormaechei* | 100 | |
| 23-2220 | 2023 | 173 | Jalisco | Secretion | No | *E. hormaechei* | 100 | |
| 23-2599 | 2023 | 78 | Sonora | Urine | No | *E. hormaechei* | 100 | |
| 23-2610 | 2023 | 78 | Sonora | Urine | No | *E. hormaechei* | 100 | |
| 23-2632 | 2023 | 78 | Sonora | Urine | No | *E. hormaechei* | 100 | |
| 23-3133 | 2023 | 78 | Sonora | Urine | No | *E. hormaechei* | 100 | |
| 23-3135 | 2023 | 78 | Sonora | Urine | No | *E. hormaechei* | 100 | |
| 24-4038 | 2024 | 78 | Sonora | Urine | No | *E. hormaechei* | 100 | |
| EB9394 | 2024 | 78 | Mexico City | Biopsy | Yes | *E. hormaechei* | 100 | |
| EB9512 | 2024 | 78 | Mexico City | Respiratory | No | *E. hormaechei* | 100 | |
| EB9845 | 2024 | 78 | Mexico City | Urine | No | *E. hormaechei* | 100 | |
| ***E. hormaechei* subsp. *hormaechei*** | | | | | | | |  |
| 20-1753 | 2020 | 528 | Mexico City | Blood | Yes | *E. hormaechei* | 100 | |
| 22-1828 | 2022 | 1848 | Coahuila | Blood | Yes | *E. hormaechei* | 100 | |
| 23-2139 | 2023 | 1749 | Sonora | Secretion | Yes | *E. hormaechei* | 98 | |
| ***E. hormaechei* subsp. *oharae*** | | | | | | | |  |
| 20-1869 | 2020 | 68 | Sonora | Urine | No | *E. hormaechei* | 100 | |
| ***E. cloacae*** | | | | | | | |  |
| 20-314 | 2020 | 3389 | Mexico City | Wound | No | *E. cloacae* | 100 | |
| 22-1332 | 2022 | 456 | Jalisco | Urine | No | *E. cloacae* | 100 | |
| 23-2461 | 2023 | 456 | Jalisco | Urine | No | *E. cloacae* | 100 | |
| 23-3076 | 2023 | 456 | Jalisco | Urine | No | *E. cloacae* | 100 | |
| 24-3369 | 2024 | 456 | Jalisco | Urine | No | *E. cloacae* | 100 | |
| 24-3602 | 2024 | 3399 | Mexico City | Pleural | Yes | *E. cloacae* | 100 | |
| ***E. asburiae*** | | | | | | | |  |
| 20-035 | 2020 | 24 | Jalisco | Wound | No | *E. asburiae* | 100 | |
| 20-1962 | 2020 | 162 | Guanajuato | Blood | Yes | *E. asburiae* | 100 | |
| 20-1965 | 2020 | 733 | Guanajuato | Blood | Yes | *E. asburiae* | 100 | |
| 22-1849 | 2022 | 3398 | Mexico State | Blood | Yes | *E. asburiae* | 100 | |
| 23-2207 | 2023 | 25 | Jalisco | Blood | Yes | *E. asburiae* | 100 | |
| **Novel Species** | | | | | | | |  |
| 20-2045 | 2020 | 3388 | Mexico City | Biopsy | Yes | *E. kobei* | 16 | |
| 20-2046 | 2020 | 3388 | Mexico City | Drainage | Yes | *E. kobei* | 16 | |
| 20-2064 | 2020 | 3395 | Mexico City | Biopsy | Yes | *E. kobei* | 18 | |
| 20-2065 | 2020 | 3388 | Mexico City | Drainage | Yes | *E. kobei* | 16 | |
| ***E. roggenkampii*** | | | | | | | |  |
| 20-1325-3 | 2020 | 2271 | Oaxaca | Urine | No | *E. roggenkampii* | 100 | |
| 20-1410 | 2020 | 2271 | Nuevo Leon | Urine | No | *E. roggenkampii* | 100 | |
| 20-1512 | 2020 | 272 | Guanajuato | Blood | Yes | *E. roggenkampii* | 100 | |
| 21-830 | 2021 | 826 | Michoacan | Wound | No | *E. roggenkampii* | 100 | |
| ***E. kobei*** | | | | | | | |  |
| 21-1117 | 2021 | 54 | Jalisco | Blood | Yes | *E. kobei* | 100 | |
| 21-1121 | 2021 | 54 | Jalisco | Blood | Yes | *E. kobei* | 100 | |
| ***E. ludwigii*** | | | | | | | |  |
| 22-2027 | 2022 | 3041 | Mexico City | Urine | No | *E. ludwigii* | 100 | |
| ***E. bugandensis*** | | | | | | | |  |
| 24-3972 | 2024 | 2268 | Jalisco | Urine | No | *E. bugandensis* | 96 | |

**Supplementary Table 3.** Quality statistics for the generated assemblies.

| Genome | # contigs | Largest contig (bp) | Total length (bp) | GC (%) | N50 (bp) | N90 (bp) | L50 | L90 | Completeness (%) | Contamination (%) |
| --- | --- | --- | --- | --- | --- | --- | --- | --- | --- | --- |
| 20-035 | 75 | 338730 | 5092870 | 55.47 | 206769 | 46464 | 10 | 28 | 99.97 | 1.42 |
| 20-053 | 65 | 697907 | 4891414 | 55.22 | 284416 | 61927 | 6 | 20 | 99.77 | 0.49 |
| 20-083 | 64 | 806427 | 4813826 | 55.53 | 209939 | 48838 | 6 | 25 | 99.47 | 0.23 |
| 20-1013 | 108 | 313180 | 5077061 | 55.26 | 143762 | 32088 | 13 | 37 | 97.39 | 0.59 |
| 20-1062 | 44 | 873421 | 4623961 | 55.48 | 237635 | 76261 | 6 | 19 | 99.47 | 0.39 |
| 20-1174 | 34 | 869698 | 4876698 | 55.23 | 456159 | 119145 | 4 | 11 | 99.77 | 0.33 |
| 20-1239 | 56 | 1026291 | 4806292 | 55.18 | 247142 | 60942 | 6 | 21 | 99.97 | 0.91 |
| 20-1270 | 50 | 475011 | 4569796 | 55.43 | 230015 | 85405 | 8 | 18 | 99.77 | 0.14 |
| 20-1324 | 134 | 366572 | 5102263 | 55.2 | 125689 | 24137 | 12 | 47 | 99.47 | 0.27 |
| 20-1325-1 | 152 | 351252 | 5364276 | 55.07 | 136232 | 24540 | 13 | 48 | 99.52 | 0.55 |
| 20-1325-2 | 27 | 1312538 | 4859321 | 55.52 | 749616 | 111105 | 3 | 10 | 99.47 | 0.35 |
| 20-1325-3 | 40 | 1195570 | 5102266 | 55.78 | 378601 | 111141 | 5 | 14 | 99.07 | 1.25 |
| 20-1395 | 56 | 827133 | 4956294 | 55.5 | 212540 | 71977 | 6 | 20 | 99.47 | 1.28 |
| 20-1410 | 38 | 1177554 | 5096456 | 55.77 | 378601 | 105715 | 4 | 13 | 99.07 | 1.25 |
| 20-1490 | 34 | 1115506 | 4913452 | 55.43 | 483398 | 113754 | 3 | 11 | 99.47 | 0.35 |
| 20-1512 | 67 | 760090 | 5023821 | 55.63 | 191030 | 47586 | 8 | 24 | 98.48 | 0.67 |
| 20-1528 | 40 | 1427822 | 4763761 | 55.49 | 349193 | 74701 | 4 | 14 | 99.21 | 0.89 |
| 20-1753 | 88 | 401890 | 4702470 | 55.22 | 177892 | 40342 | 10 | 31 | 99.97 | 0.73 |
| 20-1869 | 54 | 844380 | 4805047 | 55.25 | 226309 | 62453 | 6 | 21 | 99.62 | 0.51 |
| 20-195 | 24 | 1312764 | 4859724 | 55.53 | 652065 | 115092 | 3 | 8 | 99.47 | 0.35 |
| 20-1962 | 73 | 341428 | 4896180 | 55.67 | 146732 | 55570 | 12 | 33 | 99.97 | 0.67 |
| 20-1965 | 22 | 1755308 | 4594832 | 55.81 | 808353 | 186810 | 2 | 8 | 99.07 | 0.33 |
| 20-202 | 59 | 1119737 | 4919535 | 54.99 | 274290 | 65827 | 5 | 17 | 99.87 | 0.22 |
| 20-2031 | 32 | 736153 | 4600131 | 55.44 | 456713 | 119209 | 4 | 12 | 99.77 | 0.04 |
| 20-2045 | 42 | 586200 | 5004994 | 55.53 | 203984 | 87587 | 8 | 23 | 99.67 | 0.41 |
| 20-2046 | 33 | 640860 | 4879865 | 55.58 | 245262 | 98455 | 6 | 19 | 97.29 | 0.3 |
| 20-2063 | 61 | 779679 | 5233217 | 54.74 | 362658 | 55773 | 5 | 19 | 99.77 | 1.57 |
| 20-2064 | 27 | 1312618 | 4706316 | 55.89 | 246957 | 114792 | 4 | 14 | 99.67 | 0.04 |
| 20-2065 | 36 | 953229 | 4920896 | 55.58 | 209942 | 89180 | 7 | 21 | 99.37 | 0.3 |
| 20-223 | 60 | 579713 | 4869708 | 55.03 | 274109 | 75035 | 6 | 17 | 99.87 | 0.22 |
| 20-257 | 55 | 1156979 | 4957413 | 55.5 | 212667 | 77364 | 5 | 19 | 99.47 | 1.28 |
| 20-258 | 53 | 512482 | 4646666 | 55.3 | 303073 | 80052 | 6 | 17 | 99.77 | 0.49 |
| 20-309 | 29 | 1312626 | 4853574 | 55.52 | 1260119 | 99363 | 2 | 11 | 99.47 | 0.35 |
| 20-314 | 33 | 496183 | 5119474 | 54.57 | 317140 | 89179 | 7 | 17 | 100 | 0.87 |
| 20-485 | 54 | 1407731 | 5012546 | 54.81 | 373577 | 83881 | 4 | 14 | 99.84 | 0.65 |
| 20-500 | 73 | 759788 | 4961397 | 55.31 | 204372 | 49732 | 7 | 24 | 99.37 | 0.65 |
| 20-555 | 37 | 956950 | 4728972 | 55.47 | 468291 | 103672 | 4 | 11 | 99.77 | 0.04 |
| 21-045 | 50 | 1084266 | 4865469 | 55.54 | 497904 | 100693 | 4 | 13 | 99.47 | 1.64 |
| 21-055 | 120 | 352367 | 5213348 | 54.75 | 102106 | 30276 | 15 | 51 | 99.3 | 1.13 |
| 21-060 | 85 | 662395 | 5180221 | 55.23 | 195376 | 59691 | 8 | 26 | 99.77 | 1.76 |
| 21-065 | 79 | 709796 | 5167973 | 55.26 | 235963 | 67135 | 8 | 22 | 99.77 | 1.76 |
| 21-1013 | 44 | 1520785 | 4754336 | 55.15 | 293359 | 103755 | 4 | 15 | 99.4 | 0.76 |
| 21-1033 | 41 | 1520785 | 4754321 | 55.15 | 435160 | 107206 | 3 | 13 | 99.47 | 0.76 |
| 21-1069 | 109 | 588980 | 5401159 | 54.91 | 258856 | 40130 | 8 | 26 | 99.47 | 1.04 |
| 21-1072 | 77 | 444645 | 5032542 | 55.12 | 245953 | 53898 | 8 | 25 | 99.69 | 0.6 |
| 21-1115 | 68 | 1067104 | 4980222 | 55.13 | 269183 | 52649 | 5 | 18 | 99.92 | 0.5 |
| 21-1117 | 90 | 333764 | 5099911 | 54.63 | 150887 | 36843 | 12 | 35 | 99.7 | 1.87 |
| 21-1121 | 91 | 333712 | 5097474 | 54.63 | 140820 | 36843 | 13 | 35 | 99.7 | 1.87 |
| 21-135 | 77 | 527680 | 4937386 | 55.14 | 297191 | 49972 | 7 | 23 | 99.69 | 1.01 |
| 21-136 | 77 | 527676 | 4968650 | 55.1 | 313861 | 53898 | 6 | 21 | 99.77 | 1.01 |
| 21-233 | 37 | 825417 | 4751476 | 55.64 | 292969 | 111013 | 5 | 15 | 99.47 | 0.2 |
| 21-238 | 67 | 712059 | 4940164 | 55.34 | 166175 | 51400 | 9 | 28 | 99.47 | 0.33 |
| 21-253 | 43 | 1034456 | 4619610 | 55.33 | 218793 | 103790 | 5 | 16 | 99.77 | 0.43 |
| 21-266 | 30 | 928838 | 4764513 | 55.58 | 411522 | 115029 | 4 | 13 | 99.47 | 0.52 |
| 21-293 | 42 | 907824 | 4913993 | 55.52 | 341820 | 111035 | 5 | 14 | 99.47 | 0.27 |
| 21-326 | 84 | 539577 | 5039349 | 54.96 | 240688 | 56469 | 7 | 23 | 99.87 | 0.82 |
| 21-606 | 65 | 1067107 | 4980097 | 55.13 | 269148 | 54897 | 6 | 19 | 99.92 | 0.5 |
| 21-613 | 27 | 1162863 | 4883734 | 55.47 | 464823 | 110933 | 4 | 13 | 99.77 | 0.43 |
| 21-614 | 52 | 1002709 | 4646745 | 55.19 | 286013 | 65827 | 5 | 18 | 99.87 | 0.33 |
| 21-640-1 | 74 | 333452 | 4690276 | 55.15 | 135928 | 49603 | 11 | 31 | 99.82 | 0.53 |
| 21-640-2 | 77 | 333453 | 4692050 | 55.15 | 135926 | 45997 | 11 | 31 | 99.77 | 0.53 |
| 21-782 | 31 | 733732 | 4850158 | 55.48 | 301337 | 82719 | 5 | 16 | 99.77 | 0.1 |
| 21-830 | 84 | 314232 | 4779819 | 56 | 139342 | 38088 | 12 | 35 | 98.78 | 0.48 |
| 22-1152 | 39 | 744273 | 4555807 | 55.47 | 230055 | 99622 | 6 | 17 | 99.92 | 0.04 |
| 22-1299 | 66 | 1067108 | 4980926 | 55.13 | 326675 | 66048 | 4 | 17 | 99.84 | 0.5 |
| 22-1321 | 69 | 1067125 | 4983880 | 55.13 | 326672 | 54897 | 4 | 17 | 99.84 | 0.5 |
| 22-1326 | 65 | 660725 | 4980000 | 55.13 | 421781 | 74264 | 5 | 17 | 99.92 | 0.5 |
| 22-1330 | 69 | 1067109 | 4979474 | 55.13 | 326672 | 54897 | 4 | 18 | 99.84 | 0.5 |
| 22-1332 | 122 | 258122 | 5513784 | 54.4 | 101556 | 33021 | 18 | 50 | 100 | 1.74 |
| 22-1711 | 67 | 725716 | 4983246 | 55.13 | 479189 | 74264 | 4 | 16 | 99.77 | 0.5 |
| 22-1769 | 98 | 1040412 | 5268705 | 54.74 | 225844 | 40280 | 5 | 25 | 99.77 | 1.11 |
| 22-1772 | 126 | 529333 | 5082086 | 54.73 | 193320 | 32231 | 9 | 32 | 99.77 | 1.08 |
| 22-1790 | 143 | 298992 | 5175217 | 54.47 | 112465 | 23555 | 16 | 52 | 99.97 | 0.69 |
| 22-1795 | 111 | 676719 | 5215511 | 54.75 | 136928 | 28822 | 10 | 40 | 99.47 | 1.31 |
| 22-1828 | 53 | 923759 | 4836634 | 55.16 | 354470 | 80202 | 4 | 15 | 99.97 | 0.88 |
| 22-1849 | 24 | 1024584 | 4539158 | 56.02 | 502981 | 141759 | 3 | 10 | 99.97 | 0.33 |
| 22-1872 | 48 | 975497 | 4907020 | 55.36 | 267300 | 84560 | 5 | 17 | 99.47 | 0.33 |
| 22-2027 | 23 | 875914 | 4851389 | 54.38 | 487259 | 105399 | 4 | 12 | 99.97 | 0.14 |
| 22-2116 | 117 | 697907 | 5225395 | 54.89 | 166739 | 32895 | 9 | 32 | 99.69 | 1.4 |
| 23-2139 | 115 | 617369 | 5038298 | 54.67 | 174371 | 31175 | 9 | 34 | 99.97 | 0.19 |
| 23-2207 | 85 | 388091 | 4889897 | 55.74 | 150729 | 42829 | 11 | 33 | 99.97 | 0.63 |
| 23-2220 | 53 | 423787 | 4666196 | 55.19 | 189641 | 56175 | 9 | 26 | 99.87 | 0.29 |
| 23-2334 | 74 | 804859 | 4988405 | 55.38 | 232559 | 39562 | 7 | 24 | 99.47 | 0.67 |
| 23-2336 | 78 | 669923 | 5000291 | 54.84 | 189439 | 58701 | 7 | 25 | 99.1 | 0.51 |
| 23-2374 | 119 | 324955 | 5177486 | 55.1 | 165361 | 30952 | 12 | 37 | 99.47 | 0.33 |
| 23-2461 | 129 | 275029 | 5528676 | 54.4 | 100537 | 31126 | 16 | 51 | 100 | 1.74 |
| 23-2599 | 104 | 575962 | 5194152 | 54.78 | 232969 | 48130 | 8 | 29 | 99.87 | 0.32 |
| 23-2610 | 109 | 575925 | 5039716 | 54.9 | 187248 | 48130 | 10 | 32 | 99.87 | 0.14 |
| 23-2632 | 95 | 509394 | 5044304 | 54.93 | 196156 | 38280 | 9 | 32 | 99.87 | 0.25 |
| 23-2638 | 118 | 665504 | 5135312 | 55.17 | 153815 | 28777 | 10 | 35 | 99.47 | 0.33 |
| 23-2902 | 66 | 1067225 | 4984468 | 55.13 | 326682 | 78698 | 4 | 17 | 99.84 | 0.5 |
| 23-3059 | 27 | 850611 | 4751871 | 55.59 | 265630 | 110852 | 5 | 15 | 99.47 | 0.1 |
| 23-3076 | 137 | 301191 | 5529172 | 54.4 | 100537 | 28828 | 16 | 53 | 100 | 1.74 |
| 23-3133 | 97 | 392532 | 4974100 | 54.98 | 232969 | 41550 | 8 | 27 | 99.87 | 0.04 |
| 23-3135 | 95 | 626323 | 4976361 | 54.98 | 200814 | 49388 | 7 | 25 | 99.87 | 0.04 |
| 23-3137 | 105 | 439310 | 5093919 | 55.11 | 146522 | 41348 | 11 | 36 | 99.47 | 0.33 |
| 23-3285 | 76 | 925799 | 4987890 | 55.38 | 236315 | 47941 | 6 | 23 | 99.52 | 0.67 |
| 24-3369 | 111 | 278680 | 5290766 | 54.73 | 114389 | 43054 | 16 | 45 | 100 | 0.81 |
| 24-3457 | 76 | 925799 | 5029955 | 55.38 | 236315 | 41553 | 6 | 23 | 99.52 | 0.72 |
| 24-3602 | 45 | 587059 | 4967298 | 54.82 | 211335 | 79777 | 9 | 23 | 100 | 0.21 |
| 24-3653 | 84 | 1061818 | 5192062 | 55.18 | 209083 | 59508 | 6 | 23 | 99.77 | 1.81 |
| 24-3772 | 44 | 665880 | 4751335 | 55.38 | 298674 | 103854 | 6 | 15 | 99.69 | 0.87 |
| 24-3787 | 41 | 774530 | 4788615 | 55.51 | 298884 | 86852 | 5 | 16 | 99.47 | 0.08 |
| 24-3860 | 60 | 533979 | 4947023 | 55.08 | 189354 | 67614 | 8 | 25 | 99.77 | 0.54 |
| 24-3880 | 101 | 513594 | 5105382 | 55.12 | 136859 | 31037 | 9 | 32 | 99.47 | 0.91 |
| 24-3911 | 66 | 862161 | 4941689 | 55.14 | 279347 | 53893 | 6 | 22 | 99.47 | 0.74 |
| 24-3939 | 63 | 1354046 | 4983272 | 55.13 | 660725 | 54754 | 3 | 15 | 99.84 | 0.5 |
| 24-3972 | 78 | 456381 | 5020542 | 55.75 | 155237 | 48584 | 9 | 30 | 99.97 | 0.94 |
| 24-4038 | 131 | 487825 | 5098320 | 54.92 | 149657 | 36537 | 10 | 38 | 99.87 | 0.22 |
| 24-4047 | 122 | 357971 | 5164182 | 55.1 | 110497 | 34704 | 15 | 44 | 99.47 | 0.33 |
| 24-4048 | 101 | 379264 | 5036355 | 55.12 | 151273 | 41349 | 11 | 35 | 99.47 | 0.33 |
| 24-4054 | 120 | 357971 | 5164468 | 55.1 | 146522 | 34704 | 11 | 39 | 99.47 | 0.33 |
| EB8834 | 95 | 688300 | 5060962 | 55.18 | 318209 | 53839 | 6 | 20 | 99.77 | 1.03 |
| EB8872 | 101 | 704454 | 5000796 | 55.25 | 325021 | 49972 | 5 | 19 | 99.77 | 0.85 |
| EB9005 | 122 | 506656 | 5124772 | 54.79 | 216111 | 33652 | 8 | 26 | 99.77 | 0.53 |
| EB9393 | 69 | 587364 | 4982586 | 55.23 | 301097 | 67869 | 6 | 20 | 99.77 | 0.13 |
| EB9394 | 63 | 676160 | 4803082 | 55.08 | 349088 | 57502 | 5 | 19 | 99.57 | 0.53 |
| EB9460 | 60 | 697907 | 4830849 | 55.26 | 301373 | 67503 | 5 | 16 | 99.77 | 0.61 |
| EB9512 | 81 | 492215 | 5102978 | 54.96 | 230508 | 54740 | 7 | 25 | 99.87 | 0.53 |
| EB9554 | 72 | 753040 | 5176017 | 55.18 | 410059 | 74494 | 5 | 19 | 99.77 | 0.43 |
| EB9577 | 70 | 697907 | 4878730 | 55.25 | 301373 | 67503 | 5 | 16 | 99.77 | 0.61 |
| EB9845 | 62 | 676160 | 4803146 | 55.08 | 274120 | 65827 | 6 | 19 | 99.57 | 0.53 |

**Supplementary Table 4.** Antibiotic susceptibility profile of the *Enterobacter* isolates included in the study.

| Antibiotic | Resistant (%) | Susceptible (%) | Intermediate (%) | Non-Susceptibility (%) |
| --- | --- | --- | --- | --- |
| Tobramycin | 52.5 | 38.3 | 8.3 | 60.8 |
| Amikacin | 50.8 | 49.2 | 0.0 | 50.8 |
| Gentamicin | 47.5 | 48.3 | 3.3 | 50.8 |
| Ertapenem | 58.3 | 30.0 | 11.7 | 70.0 |
| Meropenem | 45.0 | 51.7 | 3.3 | 48.3 |
| Imipenem | 42.5 | 55.8 | 1.7 | 44.2 |
| Ceftazidime | 73.3 | 26.7 | 0.8 | 74.2 |
| Cefotaxime | 68.3 | 30.8 | 0.8 | 69.2 |
| Cefepime | 54.2 | 36.7 | 9.2 | 63.3 |
| Aztreonam | 65.8 | 33.3 | 0.0 | 65.8 |
| Piperacillin-tazobactam | 78.3 | 21.7 | 0.0 | 78.3 |
| Ceftazidime-avibactam | 41.7 | 57.5 | 0.0 | 41.7 |
| Ciprofloxacin | 59.2 | 35.0 | 5.0 | 64.2 |
| Levofloxacin | 39.2 | 54.2 | 5.8 | 45.0 |
| Trimethoprim-Sulfamethoxazole | 53.3 | 46.7 | 0.0 | 53.3 |
| Colistin | 10.8 | 0.0 | 87.5 | 98.3 |

**Supplementary Table 5.** Antibiotic susceptibility profiles, mMIC, eMIC, resistance gene screening, and multilocus sequence typing (MLST) of the included isolates.

| Isolate | MLST | TZP | CAZ | CZA | CTX | FEP | ATM | ETP | IPM | MEM | AN | CIP* | TGC* | LVX* | GM* | TM* | SXT* | CL* | mMIC | eMIC | Carbapenemases (ABRicate) |
| --- | --- | --- | --- | --- | --- | --- | --- | --- | --- | --- | --- | --- | --- | --- | --- | --- | --- | --- | --- | --- | --- |
| ***E. hormaechei* subsp. *steigerwaltii*** | | | | | | | | | | | | | | | | | | | | | |
| 20-1528 | 45 | <4 | <0.125 | 0.25 | <0.25 | <0.125 | <1 | <0.125 | <0.5 | <0.25 | 4 | <0.25 | **>8** | **24** | 16 | 15 | **6** | **4** | N/T | N/T | NEG |
| 20-500 | 45 | <4 | 0.25 | 0.25 | <0.25 | <0.125 | <1 | <0.125 | <0.25 | <0.25 | <1 | <0.062 | <0.5 | 29 | 20 | 18 | 20 | <2 | N/T | N/T | NEG |
| 21-045 | 45 | **>128** | **>64** | **>16** | **>32** | **>32** | <1 | **>8** | **>16** | **>16** | **32** | **1** | 0.5 | 20 | **6** | **6** | **6** | <2 | POS | POS | *bla*_NDM-1_ |
| 23-2334 | 45 | **>128** | **>64** | **>16** | **>32** | **>32** | **>64** | **>8** | **>16** | **>16** | **32** | **>4** | **>8** | **6** | **6** | **6** | **6** | <2 | POS | POS | *bla*_NDM-1_ |
| 23-3285 | 45 | **>128** | **>64** | **>16** | **>32** | **>32** | **>64** | **>8** | **>16** | **>16** | **32** | **>4** | **2** | **6** | **6** | **6** | **6** | <2 | POS | POS | *bla*_NDM-1_ |
| 24-3457 | 45 | **>128** | **>64** | **>16** | **>32** | **>32** | **>64** | **>8** | **>16** | **>16** | **32** | **>4** | **1** | **6** | **6** | **6** | **6** | 0.125 | POS | POS | *bla*_NDM-1_ |
| 24-3787 | 50 | **>128** | **>64** | 1 | **16** | **>32** | **>64** | 1 | 0.5 | <0.25 | <1 | 0.5 | **>8** | 24 | 22 | **20** | 18 | <2 | N/T | N/T | NEG |
| 20-1490 | 89 | <4 | 0.5 | 0.125 | <0.125 | <0.125 | <1 | <0.125 | 0.5 | <0.25 | <1 | <0.062 | <0.5 | 25 | 18 | 18 | 23 | <2 | N/T | N/T | NEG |
| 20-1013 | 91 | **>128** | **>64** | 2 | **>32** | **>32** | **>64** | **>8** | 1 | 1 | **>64** | **>4** | **>8** | **6** | **6** | **6** | **6** | <2 | NEG | N/T | NEG |
| 20-1324 | 91 | **>128** | **>64** | **>16** | **>32** | **>32** | **>64** | **>8** | 1 | **4** | **>64** | **>4** | **2** | **6** | **6** | **6** | **6** | <2 | NEG | N/T | NEG |
| 20-1325 | 91 | **>128** | **>64** | **>16** | **>32** | **>32** | **>64** | **>8** | 1 | 2 | **>64** | **4** | **2** | **6** | **13** | **11** | **6** | 0.5 | NEG | N/T | NEG |
| 23-2374 | 93 | **>128** | 8 | 0.5 | 2 | 4 | 2 | **>8** | 2 | **4** | **32** | **<4** | **1** | **6** | **6** | 14 | 21 | <2 | POS | NEG | *bla*_OXA-181_ |
| 23-2638 | 93 | **>128** | **>64** | 2 | **>32** | **>32** | **>64** | **2** | 0.5 | <0.25 | **32** | **>4** | **1** | **10** | **6** | 14 | 20 | <2 | NEG | N/T | NEG |
| 23-3137 | 93 | **>128** | **>64** | 2 | **>32** | **>32** | **>64** | **2** | 1 | <0.25 | **32** | **>4** | **1** | **6** | 18 | 18 | 21 | <2 | NEG | N/T | NEG |
| 24-4047 | 93 | **>128** | 4 | 2 | 2 | 2 | <1 | **>8** | 2 | **4** | **32** | **>4** | **1** | **6** | **6** | **13** | 22 | <2 | POS | NEG | *bla*_OXA-181_ |
| 24-4048 | 93 | **>128** | **32** | 2 | **8** | 4 | 4 | **>8** | 1 | **4** | **32** | **>4** | **1** | **6** | 17 | **17** | 19 | <2 | POS | NEG | *bla*_OXA-181_ |
| 24-4054 | 93 | **>128** | 1 | 1 | 1 | 1 | 1 | **>8** | 1 | 1 | **32** | **>4** | **1** | **6** | **6** | **13** | 22 | <2 | POS | NEG | *bla*_OXA-181_ |
| 20-1325-2 | 116 | **>128** | **>64** | 0.5 | **>32** | 4 | **>64** | 1 | <0.25 | <0.25 | 2 | <0.062 | <0.5 | 29 | 20 | 20 | 26 | 0.5 | N/T | N/T | NEG |
| 20-195 | 116 | **>128** | **<64** | 0.5 | **>32** | 4 | **>64** | **2** | <0.25 | <0.25 | 1 | <0.062 | <0.5 | 28 | 20 | 18 | 23 | <2 | NEG | N/T | NEG |
| 20-309 | 116 | **>128** | **>64** | 0.5 | **>32** | 4 | **>64** | 1 | <0.25 | <0.25 | 2 | <0.062 | <0.5 | 29 | 21 | 18 | 25 | <2 | N/T | N/T | NEG |
| 21-266 | 116 | **>128** | **>64** | 0.5 | **>32** | 2 | **>64** | 1 | 0.5 | <0.25 | 1 | <0.062 | <0.5 | 34 | 24 | 21 | 24 | <2 | N/T | N/T | NEG |
| 22-1872 | 116 | **>128** | 0.5 | 0.25 | <0.25 | <0.125 | <1 | <0.25 | <0.5 | 1 | 2 | <0.5 | <0.5 | 26 | 20 | 20 | **6** | <2 | N/T | N/T | NEG |
| 21-1069 | 133 | **>128** | **>64** | **>16** | **>32** | **>32** | **>64** | **>8** | **>16** | **>16** | **32** | **>4** | <0.5 | 25 | **6** | **6** | **6** | <2 | POS | POS | *bla*_NDM-1_ |
| 22-1795 | 146 | **>128** | **>64** | **>16** | **>32** | 8 | **16** | **>8** | **8** | **16** | <1 | <0.5 | <0.5 | 24 | 20 | 21 | **6** | <2 | POS | POS | *bla*_NDM-1_ *mcr9.1* |
| 21-055 | 184 | **>128** | **>64** | 0.5 | **>32** | **>32** | **>64** | **>8** | 1 | 1 | **>64** | **>4** | <0.5 | 6 | 18 | 18 | **6** | <2 | NEG | NEG | *mcr9.1* |
| 20-083 | 346 | **>128** | **>64** | 2 | **>32** | **>32** | **>64** | **4** | 0.5 | <0.25 | 2 | <0.062 | <0.5 | 28 | 19 | 18 | 22 | <2 | NEG | N/T | NEG |
| 20-1395 | 426 | <4 | 0.5 | 0.25 | <0.125 | <0.125 | <1 | <0.125 | 0.5 | <0.125 | <1 | <0.062 | <0.5 | 25 | 18 | 14 | 23 | <2 | N/T | N/T | NEG |
| 20-257 | 426 | <4 | 0.5 | 0.25 | <0.25 | <0.125 | <1 | <0.125 | 1 | <0.25 | <1 | <0.062 | <0.5 | 33 | 19 | 18 | 20 | <2 | N/T | N/T | NEG |
| 24-3880 | 488 | **>128** | **>64** | **>16** | **>32** | **>32** | **>64** | **>8** | **>16** | **>16** | <0.062 | **>4** | 0.5 | 25 | **6** | 6 | 18 | <2 | POS | POS | *bla*_NDM-1_ |
| 21-238 | 536 | **>128** | **>64** | 0.5 | **>32** | 4 | **>64** | **2** | <0.25 | <0.25 | 2 | <0.062 | <0.5 | 31 | 22 | 20 | 27 | <2 | NEG | N/T | *mcr9.1* |
| 20-1062 | 604 | <4 | 0.25 | 0.25 | <0.25 | <0.125 | <1 | <0.125 | <0.25 | <0.25 | 2 | <0.062 | <0.5 | 31 | 19 | 17 | 20 | <2 | N/T | N/T | NEG |
| 23-3059 | 972 | <4 | <0.125 | 0.25 | <0.25 | <0.125 | <1 | <0.125 | <0.25 | <0.25 | <1 | <0.062 | <0.5 | 34 | 21 | 18 | 24 | <2 | N/T | N/T | NEG |
| 21-233 | 981 | **>128** | **>64** | 0.5 | **8** | 0.5 | **32** | 0.25 | 0.5 | <0.25 | <1 | <0.062 | <0.5 | 33 | 21 | 21 | 24 | <2 | N/T | N/T | NEG |
| 21-293 | 3390 | <4 | 0.5 | 0.25 | <0.25 | <0.125 | <1 | <0.125 | 0.5 | <0.25 | <1 | <0.062 | <0.5 | 31 | 21 | 19 | 23 | <2 | N/T | N/T | NEG |
| 21-613 | 3391 | **>128** | **>64** | 0.5 | **>32** | 4 | **>64** | 1 | <0.25 | <0.25 | <1 | <0.062 | <0.5 | 29 | 20 | 20 | 24 | <2 | N/T | N/T | NEG |
| 21-782 | 3392 | <4 | 0.5 | 0.25 | <0.25 | <0.125 | <1 | <0.125 | 0.5 | <0.25 | 2 | <0.062 | <0.5 | 34 | 21 | 20 | 24 | <2 | N/T | N/T | NEG |
| 24-3911 | 3393 | **>128** | **>64** | **>16** | **>32** | **>32** | **>64** | **>8** | **>16** | **>16** | **32** | **>4** | **>8** | **6** | **6** | 6 | **6** | <2 | POS | POS | *bla*_NDM-1_ |
| 21-060 | 3396 | **>128** | **>64** | **>16** | **>32** | **>32** | **>64** | **>8** | **>16** | **>16** | **32** | **>4** | **>8** | **6** | **6** | **6** | **6** | <2 | POS | POS | *bla*_NDM-1_ |
| 21-065 | 3396 | **>128** | **>64** | **>16** | **>32** | **>32** | **>64** | **>8** | **>16** | **>16** | **32** | **>4** | **4** | **6** | **6** | **6** | **6** | <2 | POS | POS | *bla*_NDM-1_ |
| 24-3653 | 3396 | **>128** | **>64** | **>16** | **>32** | **>32** | **>64** | **>8** | **>16** | **>16** | **32** | **>4** | **4** | **6** | **6** | 6 | **6** | <2 | POS | POS | *bla*_NDM-1_ |
| ***E. hormaechei* subsp. *xiangfangesis*** | | | | | | | | | | | | | | | | | | | | | |
| 20-1270 | 63 | **>128** | **>64** | 0.5 | **>32** | **>32** | **>64** | **2** | 1 | <0.25 | <1 | **1** | **1** | 20 | 20 | 20 | 20 | <2 | N/T | N/T | NEG |
| 21-1115 | 92 | **>128** | **>64** | **>16** | **>32** | **>32** | **>64** | **>8** | **>16** | **>16** | **32** | **>4** | 0.5 | 25 | **6** | **6** | **6** | <2 | POS | POS | *bla*_NDM-1_ |
| 21-606 | 92 | **>128** | **>64** | **>16** | **>32** | **>32** | **>64** | **>8** | **>16** | **>16** | **32** | **>4** | <0.5 | 24 | **6** | **6** | **6** | <2 | POS | POS | *bla*_NDM-1_ |
| 22-1299 | 92 | **>128** | **>64** | **>16** | **>32** | **>32** | **>64** | **>8** | **>16** | **>16** | **32** | **>4** | 0.5 | 26 | **6** | **6** | **6** | <2 | POS | POS | *bla*_NDM-1_ |
| 22-1321 | 92 | **>128** | **>64** | **>16** | **>32** | **>32** | **>64** | **>8** | **>16** | **>16** | **32** | **>4** | **>8** | **16** | **6** | **6** | **6** | <2 | POS | POS | *bla*_NDM-1_ |
| 22-1326 | 92 | **>128** | **>64** | **>16** | **>32** | **>32** | **>64** | **>8** | **>16** | **>16** | **32** | **>4** | **>8** | **16** | **6** | **6** | **6** | <2 | POS | POS | *bla*_NDM-1_ |
| 22-1330 | 92 | **>128** | **>64** | **>16** | **>32** | **>32** | **>64** | **>8** | **>16** | **>16** | **32** | **>4** | <0.5 | 27 | **6** | **6** | **6** | <2 | POS | POS | *bla*_NDM-1_ |
| 22-1711 | 92 | **>128** | **>64** | **>16** | **>32** | **>32** | **>64** | **>8** | **>16** | **>16** | **>64** | **>4** | <0.5 | 26 | **6** | **6** | **6** | <2 | POS | POS | *bla*_NDM-1_ |
| 23-2902 | 92 | **>128** | **>64** | **>16** | **>32** | **>32** | **>64** | **>8** | **>16** | **>16** | **32** | **>4** | 0.5 | 26 | **6** | **6** | **6** | <2 | POS | POS | *bla*_NDM-1_ |
| 24-3939 | 92 | **>128** | **>64** | **>16** | **>32** | **>32** | **>64** | **>8** | **>16** | **>16** | **32** | **>4** | 0.5 | 26 | **6** | 6 | **6** | <2 | POS | POS | *bla*_NDM-1_ |
| 22-1772 | 98 | **>128** | **>64** | **>16** | **>32** | **>32** | **>64** | **>8** | **>16** | **>16** | **32** | 0.5 | <0.5 | 24 | **6** | **6** | **6** | <2 | POS | POS | *bla*_NDM-1_ *mcr9.1* |
| EB9393 | 109 | **>128** | **32** | **>16** | **>32** | 2 | <1 | 0.25 | **4** | **>16** | 4 | **>4** | **>8** | **15** | 19 | **12** | **6** | <2 | POS | POS | *bla*_VIM-23_ |
| EB9554 | 109 | **>128** | **>64** | **>16** | **>32** | **>32** | **>64** | **>8** | **>16** | **>16** | **32** | **>4** | **4** | **6** | **6** | **6** | **6** | <2 | POS | POS | *bla*_NDM-1_ *bla*_VIM-23_ |
| 20-2063 | 114 | **>128** | **>64** | **>16** | **>32** | **>32** | **>64** | **>8** | **>16** | **>16** | **32** | **>4** | <0.5 | 20 | **6** | **6** | **6** | <2 | POS | POS | *bla*_NDM-1_ |
| 23-2336 | 136 | **>128** | **>64** | **>16** | **>32** | **>32** | **>64** | **>8** | **>16** | **>16** | **32** | **>4** | **4** | **6** | **6** | **6** | **6** | <2 | POS | POS | *bla*_NDM-1_ |
| 20-258 | 171 | <4 | **32** | 0.25 | <0.25 | 2 | **16** | <0.125 | <0.25 | <0.25 | 4 | **>4** | <0.5 | 21 | **6** | **6** | **6** | <2 | N/T | N/T | NEG |
| 20-053 | 182 | **>128** | **>64** | **>16** | **>32** | **>32** | **>64** | **>8** | **8** | **>16** | **32** | **>4** | **>8** | **6** | **6** | **6** | **6** | <2 | POS | POS | *bla*_NDM-1_ |
| 21-1072 | 182 | **>128** | **>64** | **>16** | **>32** | **>32** | **>64** | **>8** | **>16** | **>16** | **32** | **>4** | **>8** | **6** | **6** | **6** | **6** | <2 | POS | POS | *bla*_NDM-1_ |
| 21-135 | 182 | **>128** | **>64** | **>16** | **>32** | **>32** | **>64** | **>8** | **>16** | **>16** | **32** | **>4** | **>8** | **6** | **6** | **6** | **6** | <2 | POS | POS | *bla*_NDM-1_ |
| 21-136 | 182 | **>128** | **>64** | **>16** | **>32** | **>32** | **>64** | **>8** | **>16** | **>16** | **32** | **>4** | **>8** | **6** | **6** | **6** | **6** | <2 | POS | POS | *bla*_NDM-1_ |
| 21-253 | 182 | 16 | 4 | 0.5 | 1 | <0.125 | <1 | 1 | <0.25 | <0.25 | <1 | <0.062 | <0.5 | 30 | 18 | 20 | **6** | <2 | N/T | N/T | NEG |
| 22-2116 | 182 | **>128** | **>64** | **>16** | **>32** | **>32** | **>64** | **>8** | **>16** | **>16** | **32** | **>4** | **>8** | **6** | **6** | **6** | **6** | <2 | POS | POS | *bla*_NDM-1_ |
| EB8834 | 182 | **>128** | **>64** | **>16** | **>32** | **>32** | **>64** | **>8** | **>16** | **>16** | **32** | **>4** | **>8** | **6** | **6** | **6** | **6** | <2 | POS | POS | *bla*_NDM-1_ |
| EB8872 | 182 | **>128** | **>64** | **>16** | **>32** | **>32** | **>64** | **>8** | **>16** | **>16** | **32** | **>4** | **>8** | **6** | **6** | **6** | **6** | <2 | POS | POS | *bla*_NDM-1_ |
| EB9005 | 182 | **>128** | **>64** | **>16** | **>32** | **>32** | **>64** | **>8** | **8** | **>16** | **32** | **>4** | **>8** | **6** | **6** | **6** | **6** | <2 | POS | POS | *bla*_NDM-1_ |
| EB9460 | 182 | **>128** | **>64** | **>16** | **>32** | **>32** | **>64** | **>8** | **>16** | **>16** | **32** | **>4** | **>8** | **6** | **6** | **6** | **6** | <2 | POS | POS | *bla*_NDM-1_ |
| EB9577 | 182 | **>128** | **>64** | **>16** | **>32** | **>32** | **>64** | **>8** | **8** | **>16** | **32** | **>4** | **1** | **6** | **6** | **6** | **6** | <2 | POS | POS | *bla*_NDM-1_ |
| 20-2031 | 264 | <4 | 0.5 | 0.25 | <0.25 | <0.125 | <1 | <0.125 | 1 | <0.25 | 2 | <0.062 | <0.5 | 25 | 20 | 17 | 21 | <2 | N/T | N/T | NEG |
| 20-555 | 264 | **>128** | 0.5 | 0.25 | <0.25 | <0.125 | <1 | <0.125 | 0.5 | <0.25 | <1 | <0.062 | 1 | 30 | 6 | 11 | 6 | <2 | N/T | N/T | NEG |
| 21-640 | 270 | **>128** | **>64** | 1 | **>32** | **>32** | **>64** | 0.25 | 1 | <0.25 | 4 | 0.125 | <0.5 | 30 | 20 | **12** | 23 | <2 | N/T | N/T | NEG |
| 21-640-2 | 270 | **>128** | **>64** | 1 | **>32** | **>32** | **>64** | 0.25 | 1 | <0.25 | 4 | 0.125 | <0.5 | 30 | 19 | **10** | 24 | <2 | N/T | N/T | NEG |
| 22-1769 | 418 | **>128** | **>64** | 0.5 | **8** | **>32** | **>64** | 1 | 1 | <0.25 | **32** | **>4** | **1** | **6** | **6** | **6** | **6** | <2 | N/T | N/T | *mcr9.1* |
| 20-485 | 459 | **>128** | **>64** | 1 | 4 | **>32** | **>64** | 0.25 | <0.25 | <0.25 | 4 | **>4** | **4** | **13** | 18 | **12** | **6** | <2 | N/T | N/T | NEG |
| 21-1013 | 544 | **>128** | **>64** | 0.5 | **8** | 2 | **>64** | **2** | 0.5 | <0.25 | <1 | <0.062 | <0.5 | 31 | 22 | 21 | 24 | <2 | NEG | N/T | NEG |
| 21-1033 | 544 | **>128** | **>64** | 0.5 | **8** | 4 | **>64** | **2** | 0.5 | <0.25 | <1 | <0.062 | <0.5 | 30 | 20 | 19 | 24 | <2 | NEG | N/T | NEG |
| 24-3860 | 544 | **>128** | **>64** | 0.5 | **8** | 8 | **>64** | **2** | 1 | <0.25 | <1 | **1** | <0.5 | 24 | 20 | **21** | 24 | <2 | NEG | N/T | NEG |
| 24-3772 | 1772 | <4 | 0.5 | 0.25 | <0.25 | <0.125 | <1 | <0.125 | <0.5 | <0.25 | <1 | <0.062 | <0.5 | 30 | 20 | **19** | 26 | <2 | N/T | N/T | NEG |
| 22-1152 | 2734 | **>64** | **>64** | <0.125 | 1 | 0.5 | **16** | 0.25 | 0.5 | <0.25 | <1 | <0.062 | <0.5 | 29 | 22 | 20 | 26 | <2 | N/T | N/T | NEG |
| 20-1174 | 3394 | **>128** | 0.5 | 0.25 | <0.25 | <0.125 | <1 | 1 | 1 | **4** | <1 | **1** | 0.5 | 22 | 19 | 18 | 24 | <2 | POS | NEG | *bla*_OXA-181_ |
| ***E. hormaechei* subsp. *hoffmannii*** | | | | | | | | | | | | | | | | | | | | | |
| 20-202 | 78 | **32** | **>64** | 0.5 | **16** | **>32** | **>64** | 0.25 | <0.25 | <0.25 | 4 | **>4** | <0.5 | 6 | **6** | **6** | **6** | <2 | N/T | N/T | NEG |
| 20-223 | 78 | **>128** | **>64** | **>16** | **>32** | **>32** | **>64** | **>8** | **8** | **>16** | **32** | **>4** | <0.5 | 25 | **6** | **6** | **6** | <2 | POS | POS | NEG |
| 21-326 | 78 | **>128** | **>64** | **>16** | **>32** | **>32** | **>64** | **>8** | **>16** | **>16** | **32** | **>4** | **1** | 6 | **6** | **6** | **6** | <2 | POS | POS | *bla*_NDM-1_ |
| 21-614 | 78 | **>128** | **>64** | 0.5 | **16** | 1 | **>64** | **2** | <0.25 | <0.25 | <1 | <0.062 | **1** | 31 | 21 | 19 | 26 | <2 | NEG | N/T | NEG |
| 23-2599 | 78 | **>128** | **>64** | 0.25 | **16** | **>32** | **>64** | 0.25 | 1 | <0.25 | **32** | **>4** | <0.5 | **6** | **6** | **6** | 23 | <2 | N/T | N/T | NEG |
| 23-2610 | 78 | **>128** | **>64** | 2 | **>32** | **>32** | **>64** | 1 | 0.5 | <0.25 | **32** | **>4** | **>8** | **6** | **6** | **6** | 19 | <2 | N/T | N/T | NEG |
| 23-2632 | 78 | **>128** | **>64** | 2 | **16** | **>32** | **>64** | 1 | **4** | 0.5 | **32** | **>4** | **>8** | **6** | 18 | **6** | **6** | <2 | N/T | N/T | NEG |
| 23-3133 | 78 | **>128** | **>64** | 0.5 | **>32** | **>32** | **>64** | **2** | <0.25 | <0.25 | **32** | **>4** | <0.5 | **6** | **6** | **6** | 24 | <2 | NEG | N/T | NEG |
| 23-3135 | 78 | **>128** | **>64** | 1 | **>32** | **>32** | **>64** | **4** | 0.5 | <0.25 | **32** | **4** | <0.5 | **6** | **6** | **6** | 20 | <2 | NEG | N/T | NEG |
| 24-4038 | 78 | **>128** | **>64** | 0.5 | **>32** | **>32** | **>64** | **2** | 0.5 | <0.25 | **32** | **>4** | <0.5 | **6** | **6** | 6 | **6** | <2 | NEG | N/T | NEG |
| EB9394 | 78 | **>128** | **>64** | **>16** | **>32** | **>32** | **>64** | **>8** | **8** | **>16** | **32** | **>4** | **1** | **6** | **6** | **6** | **6** | <2 | POS | POS | *bla*_NDM-1_ |
| EB9512 | 78 | **>128** | **>64** | **>16** | **>32** | **>32** | <1 | **>8** | **8** | **>16** | **32** | **>4** | **>8** | **16** | **6** | **6** | **6** | <2 | POS | POS | *bla*_NDM-1_ |
| EB9845 | 78 | **>128** | **>64** | **>16** | **>32** | **>32** | **>64** | **>8** | **8** | **>16** | **32** | **>4** | **1** | **6** | **6** | **6** | **6** | <2 | POS | POS | *bla*_NDM-1_ |
| 22-1790 | 145 | **>128** | **>64** | **>16** | **>32** | **>32** | **>64** | **>8** | **8** | **>16** | **16** | **>4** | <0.5 | 22 | **6** | **6** | 20 | <2 | POS | POS | *bla*_NDM-1_ *mcr9.1* |
| 23-2220 | 173 | **>128** | 0.5 | 0.25 | 0.5 | <0.125 | <1 | 1 | 1 | 2 | <1 | **1** | <0.5 | 20 | 21 | 20 | 26 | <2 | POS | NEG | *bla*_OXA-181_ |
| 20-1239 | 233 | 8 | 0.5 | 0.25 | <0.25 | <0.125 | <1 | <0.125 | <0.25 | <0.25 | <1 | <0.062 | <0.5 | 28 | 20 | 19 | 23 | <2 | N/T | N/T | NEG |
| ***E. hormaechei* subsp. *hormaechei*** | | | | | | | | | | | | | | | | | | | | | |
| 20-1753 | 528 | **64** | **32** | 0.5 | 1 | 0.25 | **16** | 0.25 | <0.25 | <0.25 | 2 | <0.062 | <0.5 | 26 | 19 | 18 | **6** | <2 | N/T | N/T | NEG |
| 23-2139 | 1749 | **>128** | **>64** | **>16** | **>32** | **>32** | **>64** | **>8** | **>16** | **>16** | <1 | 0.5 | <0.5 | 23 | 20 | 18 | **6** | <2 | POS | POS | *bla*_NDM-1_ *mcr9.1* |
| 22-1828 | 1848 | **>128** | **>64** | 0.5 | 2 | 0.5 | **>64** | 1 | 0.5 | <0.25 | 1 | <0.062 | <0.5 | 30 | 21 | 18 | **6** | <2 | N/T | N/T | NEG |
| ***E. hormaechei* subsp. o*harae*** | | | | | | | | | | | | | | | | | | | | | |
| 20-1869 | 68 | <4 | <0.125 | <0.125 | <0.25 | <0.125 | <1 | <0.125 | <0.25 | <0.25 | 2 | <0.125 | <0.5 | 25 | 19 | 18 | 24 | <2 | N/T | N/T | NEG |
| ***E. cloacae*** | | | | | | | | | | | | | | | | | | | | | |
| 22-1332 | 456 | **>128** | **>64** | **>16** | **>32** | **>32** | **>64** | **>8** | **>16** | **>16** | **32** | **>4** | **1** | 6 | **6** | **6** | **6** | **>64** | POS | POS | *bla*_NDM-1_ |
| 23-2461 | 456 | **>128** | **>64** | **>16** | **>32** | **>32** | **>64** | **>8** | **>16** | **>16** | **32** | **>4** | **1** | **6** | **6** | **6** | **6** | **8** | POS | POS | *bla*_NDM-1_ |
| 23-3076 | 456 | **>128** | **>64** | **>16** | **>32** | **>32** | **>64** | **>8** | **>16** | **>16** | **32** | **>4** | **1** | **6** | **6** | **6** | **6** | **16** | POS | POS | *bla*_NDM-1_ |
| 24-3369 | 456 | **>128** | **>64** | **>16** | **>32** | **>32** | **>64** | **>8** | **>16** | **>16** | **32** | **>4** | **4** | **6** | **6** | **6** | **6** | 1 | NEG | N/T | *bla*_NDM-1_ |
| 20-314 | 3389 | **>128** | **>64** | 0.5 | **8** | 0.5 | **>64** | **2** | 1 | <0.25 | 1 | <0.062 | <0.5 | 28 | 19 | **9** | 24 | **64** | NEG | N/T | NEG |
| 24-3602 | 3399 | **>8** | **>16** | >16 | **32** | >4 | **1** | 6 | 6 | 6 | 6 | 0.125 | POS | POS | NDM | 0 | 0 | 0 | N/T | N/T | NEG |
| ***E. asburiae*** | | | | | | | | | | | | | | | | | | | | | |
| 20-035 | 24 | **>128** | **>64** | **>16** | **>32** | **>32** | <1 | **>8** | **8** | **8** | **32** | 0.125 | <0.5 | 30 | 19 | 19 | **6** | <2 | POS | POS | *bla*_NDM-1_ |
| 23-2207 | 25 | **>128** | **16** | 8 | **>32** | 0.5 | <1 | 0.75 | **32** | **12** | 4 | **>4** | <0.5 | 21 | 18 | **11** | 20 | 0.125 | POS | POS | *bla*_VIM-23_ |
| 20-1962 | 162 | <4 | <0.125 | <0.125 | <0.25 | <0.125 | <1 | <0.125 | <0.5 | <0.25 | <1 | <0.062 | <0.5 | 28 | 19 | **9** | 22 | **16** | N/T | N/T | NEG |
| 20-1965 | 733 | <4 | 0.25 | 0.25 | 0.25 | <0.125 | <1 | <0.125 | <0.5 | <0.25 | 2 | <0.125 | <0.5 | 25 | 20 | 18 | 22 | <2 | N/T | N/T | NEG |
| 22-1849 | 3398 | <4 | 0.25 | <0.125 | <0.25 | <0.125 | <1 | <0.125 | 1 | <0.25 | 2 | <0.062 | <0.5 | 30 | 20 | 18 | 22 | **64** | N/T | N/T | NEG |
| **Novel Species** | | | | | | | | | | | | | | | | | | | | | |
| 20-2045 | 3388 | <4 | 0.5 | 0.25 | <0.25 | <0.125 | <1 | <0.125 | <0.5 | <0.25 | <1 | <0.062 | <0.5 | 29 | 20 | 19 | 23 | **8** | N/T | N/T | *bla*_NDM-1_ |
| 20-2046 | 3388 | <4 | 0.5 | 0.25 | <0.25 | <0.125 | <1 | <0.125 | 0.5 | <0.25 | 2 | <0.062 | <0.5 | 26 | 16 | 16 | 17 | **16** | N/T | N/T | NEG |
| 20-2065 | 3388 | 8 | 0.5 | 0.25 | <0.25 | <0.125 | <1 | <0.125 | 1 | <0.25 | 2 | <0.062 | <0.5 | 27 | 19 | 17 | 24 | **64** | N/T | N/T | NEG |
| 20-2064 | 3395 | <4 | 0.25 | 0.25 | <0.25 | <0.125 | <1 | <0.125 | <0.5 | <0.25 | 4 | <0.062 | <0.5 | 29 | 16 | 18 | 22 | **>64** | N/T | N/T | NEG |
| ***E. roggenkampii*** | | | | | | | | | | | | | | | | | | | | | |
| 20-1512 | 272 | <4 | <0.125 | <0.125 | <0.25 | <0.125 | <1 | <0.125 | 0.5 | <0.25 | 4 | <0.062 | <0.5 | 34 | 19 | 18 | 21 | <2 | N/T | N/T | NEG |
| 21-830 | 826 | **>128** | **>64** | 0.5 | **16** | 1 | **>64** | 0.25 | 1 | <0.25 | <1 | <0.062 | **1** | 31 | 20 | 19 | **6** | <2 | N/T | N/T | NEG |
| 20-1325-3 | 2271 | 16 | 2 | 8 | 1 | 0.25 | <1 | <0.125 | 1 | <0.25 | <1 | **>4** | **>8** | **6** | 19 | 20 | **6** | 0.125 | N/T | N/T | NEG |
| 20-1410 | 2271 | 16 | 1 | 1 | 1 | 0.25 | <1 | <0.125 | 1 | <0.25 | <1 | **>4** | **>8** | **6** | 22 | 20 | **6** | **8** | N/T | N/T | NEG |
| ***E. kobei*** | | | | | | | | | | | | | | | | | | | | | |
| 21-1117 | 54 | **>128** | **>64** | **>16** | **>32** | 4 | <1 | 1 | **8** | **>16** | **>64** | 0.5 | **<1** | 25 | 20 | 20 | **6** | <2 | POS | POS | *bla*_NDM-1_ *mcr9.1* |
| 21-1121 | 54 | **>128** | **>64** | **>16** | **>32** | **>32** | <1 | **2** | **8** | **>16** | **32** | 0.5 | <0.5 | 24 | 19 | 19 | **6** | **16** | POS | POS | *bla*_NDM-1_ *mcr9.1* |
| ***E. ludwigii*** | | | | | | | | | | | | | | | | | | | | | |
| 22-2027 | 3041 | **>128** | 0.5 | 0.25 | 0.5 | <0.125 | <1 | **2** | 1 | 2 | 2 | **1** | 0.5 | 22 | 18 | 17 | 26 | <2 | POS | NEG | *bla*_OXA-181_ |
| ***E. bugandensis*** | | | | | | | | | | | | | | | | | | | | | |
| 24-3972 | 2268 | <4 | <0.125 | <0.125 | <0.25 | <0.125 | <1 | <0.125 | <0.25 | <0.25 | 2 | 0.5 | <0.5 | 26 | 21 | **18** | 23 | <2 | N/T | N/T | NEG |

Values in bold indicate resistance to the corresponding antibiotic. Abbreviations: TZP, piperacillin-tazobactam; CAZ, ceftazidime; CZA, ceftazidime-avibactam; CTX, cefotaxime; FEP, cefepime; ATM, aztreonam; ETP, ertapenem; IPM, imipenem; MEM, meropenem; AMK, amikacin; CIP, ciprofloxacin; TGC, tigecycline; LVX, levofloxacin; GEN, gentamicin; TOB, tobramycin; SXT, trimethoprim-sulfamethoxazole; CST, colistin; mMIC, modified carbapenem inactivation method; eMIC, mCIM in the presence of EDTA; AMRs, relevant antimicrobial resistance genes identified using ABRicate; N/T, not tested.

* Tested using the Kirby-Bauer disk diffusion method.

Tigecycline (TIG) susceptibility was interpreted according to EUCAST version 15.

**Supplementary Table 6.** Replicon types and predicted mobility of plasmids carrying relevant antimicrobial resistance determinants.

| Gene and replicon type | Conjugative | Mobilizable | Non-mobilizable | Total |
| --- | --- | --- | --- | --- |
| ***bla*_CTX-M-15_** | **23** | **2** | **28** | **53** |
| Not identified | 0 | 0 | 25 | 25 |
| IncC | 1 | 2 | 0 | 3 |
| IncC,IncFIA | 2 | 0 | 0 | 2 |
| IncFIA | 2 | 0 | 0 | 2 |
| IncFIA,IncFIC | 2 | 0 | 0 | 2 |
| IncFIB | 1 | 0 | 3 | 4 |
| IncFIB,IncFII,rep_cluster_2272 | 3 | 0 | 0 | 3 |
| IncFIB,IncHI1B | 1 | 0 | 0 | 1 |
| IncFIB,rep_cluster_2183 | 1 | 0 | 0 | 1 |
| IncFII | 1 | 0 | 0 | 1 |
| IncHI1A | 1 | 0 | 0 | 1 |
| IncHI2A,rep_cluster_1088 | 5 | 0 | 0 | 5 |
| IncI-gamma/K1 | 1 | 0 | 0 | 1 |
| IncL/M | 1 | 0 | 0 | 1 |
| IncL/M,rep_cluster_1254 | 1 | 0 | 0 | 1 |
| ***mcr9.1*** | **4** | **0** | **2** | **6** |
| Not identified | 0 | 0 | 1 | 1 |
| IncHI2A,IncR,rep_cluster_1088 | 1 | 0 | 1 | 2 |
| IncHI2A,rep_cluster_1088 | 2 | 0 | 0 | 2 |
| rep_cluster_1088 | 1 | 0 | 0 | 1 |
| ***bla*_NDM-1_** | **39** | **0** | **6** | **45** |
| Not identified | 0 | 0 | 5 | 5 |
| IncC | 1 | 0 | 0 | 1 |
| IncC,IncFIB | 1 | 0 | 0 | 1 |
| IncFIA | 0 | 0 | 1 | 1 |
| IncFIB | 1 | 0 | 0 | 1 |
| IncFIB,IncFII | 2 | 0 | 0 | 2 |
| IncFIB,IncFII,rep_cluster_2272 | 33 | 0 | 0 | 33 |
| IncFIB,IncHI1B | 1 | 0 | 0 | 1 |
| ***bla*_OXA-1_** | **10** | **0** | **1** | **11** |
| IncFIA,IncFIC | 1 | 0 | 0 | 1 |
| IncFIB | 0 | 0 | 1 | 1 |
| IncFIB,IncFII,rep_cluster_2272 | 4 | 0 | 0 | 4 |
| IncHI2A,rep_cluster_1088 | 4 | 0 | 0 | 4 |
| IncN | 1 | 0 | 0 | 1 |
| ***bla*_OXA-10_** | **2** | **0** | 0 | **2** |
| IncC | 2 | 0 | 0 | 2 |
| ***bla*_OXA-181_** | **6** | **0** | 0 | **6** |
| IncX3,rep_cluster_1195 | 6 | 0 | 0 | 6 |
| ***bla*_OXA-2_** | **0** | 0 | **2** | **2** |
| Not identified | 0 | 0 | 2 | 2 |
| ***bla*_TEM-1_** | **16** | **2** | **20** | **38** |
| Not identified | 0 | 0 | 15 | 15 |
| IncFIA,IncR | 0 | 2 | 0 | 2 |
| IncFIB | 0 | 0 | 4 | 4 |
| IncFIB,IncFII | 2 | 0 | 1 | 3 |
| IncFIB,IncFII,rep_cluster_2272 | 5 | 0 | 0 | 5 |
| IncHI2A,IncR,rep_cluster_1088 | 1 | 0 | 0 | 1 |
| IncHI2A,rep_cluster_1088 | 6 | 0 | 0 | 6 |
| IncL/M | 1 | 0 | 0 | 1 |
| rep_cluster_1088 | 1 | 0 | 0 | 1 |
| ***bla*_TEM-103_** | **2** | **2** | **3** | **7** |
| Not identified | 0 | 0 | 3 | 3 |
| IncC | 0 | 2 | 0 | 2 |
| IncFIA,IncFIC | 1 | 0 | 0 | 1 |
| IncL/M | 1 | 0 | 0 | 1 |
| ***bla*_TEM-150_** | **0** | 0 | **1** | **1** |
| Not identified | 0 | 0 | 1 | 1 |
| ***bla*_TEM-4_** | **0** | 0 | **1** | **1** |
| Not identified | 0 | 0 | 1 | 1 |
| Total | 102 | 6 | 66 | 174 |
